# Sequencing of Chinese castor lines reveals genetic signatures of selection and yield-associated loci

**DOI:** 10.1038/s41467-019-11228-3

**Published:** 2019-07-31

**Authors:** Wei Fan, Jianjun Lu, Cheng Pan, Meilian Tan, Qiang Lin, Wanfei Liu, Donghai Li, Lijun Wang, Lianlian Hu, Lei Wang, Chen Chen, Aimin Wu, Xinxin Yu, Jue Ruan, Jun Yu, Songnian Hu, Xingchu Yan, Shiyou Lü, Peng Cui

**Affiliations:** 1 Agricultural Genomics Institute, Chinese Academy of Agricultural Sciences, 518120 Shenzhen, Guangdong China; 2 CAS Key Laboratory of Plant Germplasm Enhancement and Specialty Agriculture, Wuhan Botanical Garden, Chinese Academy of Sciences, 430074 Wuhan, Hubei China; 3 University of Chinese Academy of Sciences, 100049 Beijing, China; 4 Sino-Africa Joint Research Center, Chinese Academy of Sciences, 430074 Wuhan, Hubei China; 5 Key Laboratory of Biology and Genetic Improvement of Oil Crops, Ministry of Agriculture and Rural Affairs, Oil Crops Research Institute of the Chinese Academy of Agricultural Sciences, 430062 Wuhan, Hubei China; 6 Beijing Institute of Genomics, Chinese Academy of Sciences, 100101 Beijing, China; 7 Guangdong Key Laboratory for Innovative Development and Utilization of Forest Plant Germplasm, College of Forestry and Landscape Architectures, South China Agricultural University, 510642 Guangzhou, Guangdong China; 8 State Key Laboratory of Biocatalysis and Enzyme Engineering, School of Life Sciences, Hubei University, 434200 Wuhan, Hubei China

**Keywords:** Genetic variation, Plant domestication, Natural variation in plants, Agricultural genetics

## Abstract

Oil produced by castor (*Ricinus communis*) has broad industrial applications. However, knowledge on the genetic diversity, especially genetic alterations that occurred during domestication and subsequent traits selection, of this oil crop is limited. Here, our population genomics analyses show that the Chinese castors have developed a geographic pattern, classified into the southern-, the middle-, and the northern-China groups. We detect a number of candidate genomic loci that are associated with the selection signals during the geographical differentiation and domestication. Using genome-wide association analysis, we identify candidate genes associated with nine agronomically important traits. One of the candidate genes encoding a glycosyltransferase related to cellulose and lignin biosynthesis is associated with both capsule dehiscence and endocarp thickness. We hypothesize that the abundance of cellulose or lignin in endocarp is an important factor for capsule dehiscence. Our results provide foundation for castor breeding and genetic study.

## Abstract (web summary)

Castor is an important industrial oil crop, but knowledge on its genetic diversity is limited. Here, Fan et al. show geographic pattern of Chinese castors that have developed during domestication by population genetic analyses, and reveal candidate genes associated with agronomically important traits.

## Introduction

Castor (*Ricinus communis*) is a high-oil-content herbaceous plant that is cultivated worldwide. This tropical perennial shrub, originated from Africa^1,2^, belongs to the *Euphorbiaceae* family. Its seeds contain nearly 46–55% oil by weight. The oil derivatives are widely used as basic or supplementary materials for many industrial applications, such as lubricants^3^, cosmetics, paint, coatings^4^, medicines^5^, ink and many others^6–8^. Specifically, ricinoleic acid^9^, a major component of castor oil, has special physical and chemical properties that will be extremely valuable for biodiesel in the near future^10–12^.

The wild relatives of castor are distributed in Africa, which have been cultivated for thousands of years in tropical and subtropical countries, especially in India, Brazil, and China^6^. In China, the history of castor cultivation can be traced back to ~1400 years ago according to the ancient Chinese literature (*Tang Materia Medica*). More than 1000 natural castor varieties have been grown in China, from the south to the north, which form a rich genetic resource for castor breeding. However, the domestication and population structure of castor remain largely unknown. Moreover, genome association study has not yet been performed for castor.

Taking advantage of the availability of a draft castor genome sequence^1^, we sequence 405 diverse castor accessions, which include wild varieties from Africa as well as landraces and breeding lines from 24 castor planting provinces of China. We use the identified genetic variations for genome-wide association analysis. Our results reveal several candidate genes associated with agricultural traits and a number of candidate loci that have been selected during castor domestication and geographical differentiation.

## Results

### Sequencing and phenotypic variations in all accessions

To study the domestication history of castor in China and identify the genomic loci associated with domestication, we first obtained 26 wild castors from Africa as the control group. To study the population structure of Chinese castor lines, we tried to collect samples extensively across China from south to north and obtained 376 samples from 24 castor planting provinces. In addition, considering the requirements for genome-wide association studies (GWASs), we selected samples that had enough among-accession phenotypic variations. We finally obtained 405 accessions that included 180 landraces, 199 breeding lines, and 26 wild varieties. The samples were from field-grown accessions (Wuhan, Hubei, N30°42′38.29″, E114°30′55.04″; Supplementary Data 1) and nine yield-related traits, namely, capsule dehiscence (CD), endocarp thickness (ET), hundred-grain weight (HGW), panicle height (PAH), panicle length (PAL), plant height (PH), ratio of male to female flowers (RMFF), seed length (SL), and seed volume (SV) were assessed (Supplementary Fig. 1). We performed whole-genome sequencing of these 405 accessions by Illumina HiSeq X-Ten sequencer. We obtained a total of 2.02 Tb of high-quality raw sequences, with average genome coverage of 18.8× (11.7–39.9×) and average reference genome coverage of 92.9% (88.5–94.3%) for each line after filtering the adapter and low-quality sequences. The alignments to the reference genome sequence (*R. communis* v0.1^1^) yielded 4,057,720 single-nucleotide polymorphisms (SNPs, 11.6 SNPs/kb) and 876,681 insertion–deletions (InDels, 2.5 kb). Each sample was supported by 9.5 (5.3–21.0) reads per SNP site on average (Supplementary Data 2 and Supplementary Fig. 2). Among these variations, 136,916 (3.4%) SNPs were located in coding regions, 79,523 and 57,393 of which were annotated as nonsynonymous and synonymous, respectively (Supplementary Table 1). We validated 98 SNP sites by applying Sanger sequencing (Supplementary Table 5). A total of 99.61% of the SNPs in the test were correctly detected by Sanger sequencing. This result suggested high reliability for SNP identification.

### Population structure and cultivation history

**Fig. 1:**
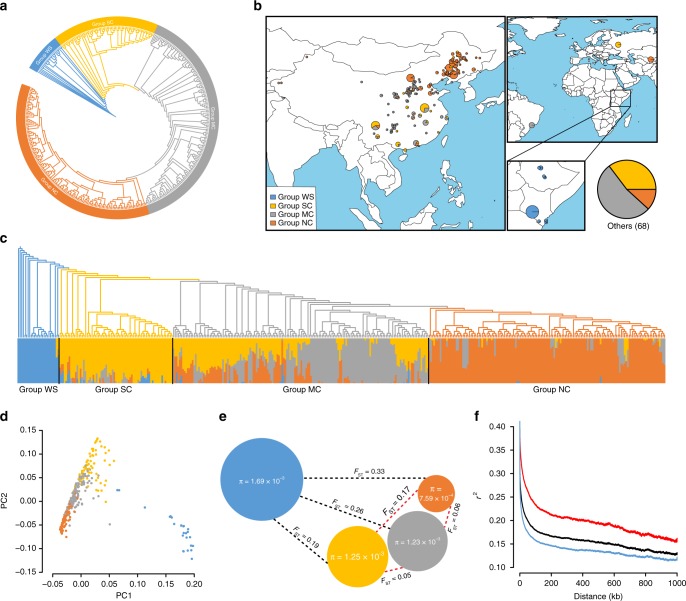
Population structure and geographic distribution of 405 castor accessions. **a** Phylogenetic tree of all accessions inferred from whole-genome single-nucleotide polymorphisms filtered by linkage disequilibrium (LD) *r*^2^ < 0.05; group names are given in the rings. **b** The geographic distribution of all accessions. The size of each pie chart represents the sample size in a specific location, with different scales shown in different frames for clarity. **c** The structure analysis (*K* = 4) matches the phylogenetic tree. The color in the structure plot indicates the component state in each sample. **d** Principal component analysis, showing the first two principal components; dot colors correspond to the phylogenetic tree grouping. **e** The diversity (*π*) and genetic distance (*F*_ST_) across the groups, where color: phylogenetic group; radius of pie: genetic diversity; and dashed line length: *F*_ST_ value between two groups. **f** LD decay line chart. Blue: group WS; black: all accessions; red: all but group WS. Source data of **c**, **d**, and **f** are provided as a Source Data file

The SNP-based phylogenetic tree (with a linkage disequilibrium (LD) threshold of *r*^2^ < 0.05) divides all accessions into four major groups (Fig. 1a, Supplementary Data 3), and the groups display clearly different geographic distributions (Fig. 1b). Group I, at the root, contains all 26 wild African accessions, the wild group (WS). The domesticated accessions, groups II (71), III (160), and IV (148), are partitioned into the southern (SC, from the coastline of southern China to the Yangtze River downstream basin), middle (MC, inland to the southeastern coastline), and northeastern groups (NC, centralized in northeastern China, with a few accessions in the Bohai Sea basin), albeit with no clear boundary between landraces and breeding lines (Supplementary Fig. 3). This result is consistent with the current situation in that the breeding of castor in China has been limited to a short period and that there is an absence of a dominant line during this period.

We further analyzed the population structure of the Chinese castor varieties based on independent SNP sites (LD *r*^2^ < 0.05) that were extracted from the whole SNP pool using ADMIXTURE (Fig. 1c and Supplementary Fig. 4). Based on the minimum coefficient of variation (CV) error value (*K* = 4), all *R. communis* accessions were grouped into four subpopulations that correlate with their geographic distribution and phylogeny. Most individuals of MC exhibit admixture, indicating intensive crossing among the accessions and the subpopulations. The principal component analysis (PCA) of all accessions is consistent with the phylogeny and the population structure (Fig. 1d).

To investigate genetic divergence among the subpopulations, we calculated the genetic distance (fixation index values, *F*_ST_) across the genome between the wild group and each cultivated group (Fig. 1e). The *F*_ST_ value (*F*_ST_ = 0.33) in the NC group is obviously larger than that in the SC group (*F*_ST_ = 0.19) and the MC group (*F*_ST_ = 0.26), and the value in the SC group is the smallest, suggesting that the NC group and the SC group populations have a furthest and closest distance to the wild population, respectively. This result is consistent with the phylogenic analysis. We also calculated the *F*_ST_ values between the wild and cultivated accessions from each province. The results show that *F*_ST_ values also display a geographically correlated pattern (Supplementary Fig. 5).

We further investigated the genetic diversity by calculating the nucleotide diversity (*π*) in each group. The genetic diversity (*π*) of the wild accessions is 1.69 × 10^−3^, which is less than that of the wild rice *Oryza rufipogon*^13^ (*π* = 3 × 10^−3^) and the wild soybean *Glycine soja*^14^ (*π* = 2.94 × 10^−3^). The domesticated groups harbor relatively little diversity, such as the SC (*π* = 1.25 × 10^−3^), the MC (*π* = 1.23 × 10^−3^), and the NC groups (*π* = 7.59 × 10^−4^). This result is consistent with the general expectation that wild populations have higher genetic diversity than domesticated populations. Interestingly, this geographic distribution displays basically the same pattern as that in a soybean study^14^, where groups 0, I, and II followed the direction from south to north and had values of 2.94 × 10^−3^, 1.44 × 10^−3^, and 1.10 × 10^−3^, respectively. Similarly, in cotton^15^, the SC, YZR, and YER groups have *π* values of 0.211 × 10^−3^, 0.197 × 10^−3^, and 0.199 × 10^−3^, respectively. In addition, the nucleotide diversity values of the Chinese castors decline from the south to the north, and the rate of reduction in genetic diversity is higher between the MC and NC groups (2-fold) than between the SC and MC groups (1.5-fold). This result suggests different intensities of selection during domestication or geographical differentiation.

Since LD decay is often associated with genetic diversity and domestication history, we further examined the mean LD values based on *r*^2^ in the different groups (Fig. 1f and Supplementary Fig. 6). The distances when dropped to half of the maximum value for the WS group and the domesticated group are 405 bp (*r*^2^ = 0.28) and 1.5 kb (*r*^2^ = 0.36), respectively. Apparently, the domesticated castor group has a relatively low rate of decline compared with the wild population. The faster drop from the maximum value in castor is distinct from that in some predominantly selfing species, such as rice (20, 123, and 167 kb in *O. rufipogon*^13^, *O. indica*, and *O. japonica*, respectively) and soybean^14^ (27, 83, and 133 kb in wild species, landraces, and improved cultivars, respectively), but similar to that in some outcrossing plants, such as maize^16^, in which the value dropped to *r*^2^ = 0.1 at 1 kb. In addition, among the SC, MC, and NC groups, LD decay is related to the geographic distribution, with a lower rate of LD decline (~761.4 kb, *r*^2^ = 0.25) in the NC group than in the SC group (47.4 kb, *r*^2^ = 0.25).

### Selections of domestication and geographical differentiation

**Fig. 2:**
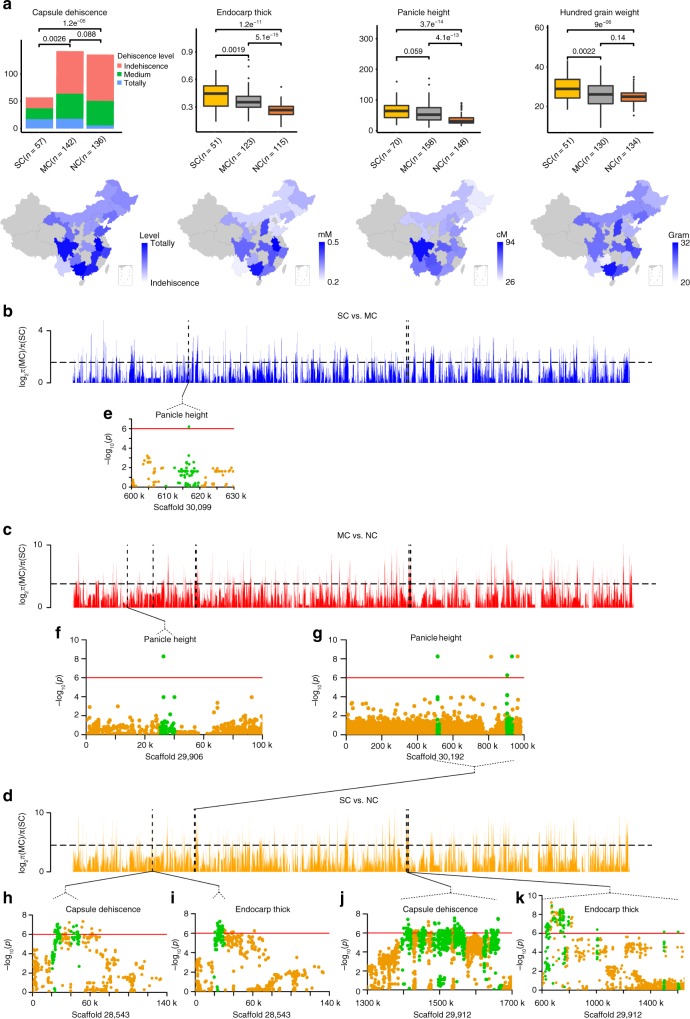
Geographical phenotype differentiation and genome regions under selection among groups. **a** Boxplot of phenotypes in group SC, MC, and NC coordinated with the heat map of phenotypes in each province in China. The center line reflects the median. The box limits are the upper and lower quartiles. Whiskers extend to data <1.5 times the interquartile range. The dots represent outliers. (*P* value shown, Wilcoxon test.) Each province in gray produced no samples or no more than three samples from the district. **b**–**d** *π* ratio bar plot with a top 5% dashed line for groups SC vs. MC, MC vs. NC, and SC vs. NC. **e**–**k** Single-nucleotide polymorphisms associated with capsule dehiscence, endocarp thickness, and panicle height by genome-wide association study merged with the selected region. Green dot: windows that underwent selection in the genome. Red cutoff line: −log_10_*P* = 6

We first found that some agricultural traits, including capsule dehiscence, endocarp thickness, hundred-grain weight, and panicle height, showed obvious differences among the three geographical groups (Fig. 2a). In addition, panicle length, plant height, and seed length exhibited differences between the NC and SC groups (Supplementary Fig. 7). Using the threshold of top 5% of the ratio of the genetic diversity (*π*) between two groups, we then found 2905 candidates by calculating genomic windows associated with the geographical groups (Supplementary Data 4). Through GWAS analysis of the 9 traits among 379 landraces or breeding lines, we identified 1487 SNPs that were significantly associated with the traits. We found that 101 GWAS signals located in 19 windows (in 2 genomic regions: 20,001–50,000 bp: scaffold 28,543; 1,390,001–1,670,000 bp: scaffold 29,912) for capsule dehiscence, 25 GWAS signals located in 1 window (in 1 genomic region: 20,001–30,000 bp: scaffold 28,543) for endocarp thickness, 133 GWAS signals located in 19 windows (in 4 genomic regions: 50,001–60,000 bp: scaffold 29,906; 620,001–1,610,000 bp: scaffold 29,912; 610,001–620,000 bp: scaffold 30,099; 510,001–930,000 bp: scaffold 30,192) for panicle height, 36 GWAS signals located in 17 windows (in 11 genomic regions distributed in scaffolds 27,699; 28,192; 28,609; 29,005; 29,579; 29,621; 29,670; 29,847; 29,915; 30,170; and 30,174) for the ratio of male to female flowers, 32 GWAS signals located in 3 windows (in 2 genomic regions: 20,001–50,000bp: scaffold 28,543; 90,001–100,000 bp: scaffold 29,876) for seed volume, and 1 GWAS signal (in 92,521 bp: scaffold 29,876) for seed length underwent selection during the course of geographical differentiation (Fig. 2b–k and Supplementary Data 5). Capsule dehiscence is a key agronomic trait that is under strong artificial selection, which is similar to the case of soybean pod shattering^17^. The capsule dehiscence trait and its underlying genes were obviously selected in the NC group, probably due to adaptation to a drier climate in northern China or selection during mass cultivation. In addition, Gene Ontology (GO) analysis revealed that 1909 genes from the genomic regions associated with geographic differentiation were enriched in several biological processes, such as the development of flowers; cell wall biogenesis; lipid, pectin, and xanthine catabolic processes; and response to hormones and stresses (Supplementary Fig. 9 and Supplementary Data 8).

We further identified selection signals associated with domestication by calculating the ratio of the genetic diversity (*π*) in wild accessions to that in domesticated accessions and the population differentiation (*F*_ST_) between the wild and domesticated lines in a 10-kb nonoverlapping window along each genome scaffold. Using the selected thresholds (top 10% of *F*_ST_ values and top 10% of *π* (wild)/*π* (landraces) values), 1258 genomic windows with selection signals were identified (Supplementary Fig. 8 and Supplementary Data 6). By alignment with GWAS signals, we found that 6 genomic regions (15 windows) were associated with panicle height and seed volume underwent selection during domestication or local breeding (Supplementary Fig. 8 and Supplementary Data 7). These results suggested that some important agricultural traits and their underlying genes underwent selection during domestication. GO analysis of 747 genes from the genomic region associated with domestication revealed that these genes are significantly enriched in several biological processes, such as protein phosphorylation, response to abscisic acid, programmed cell death, and meristem maintenance (Supplementary Fig. 9 and Supplementary Data 8).

### Candidate genes related to dehiscence and endocarp thickness

**Fig. 3:**
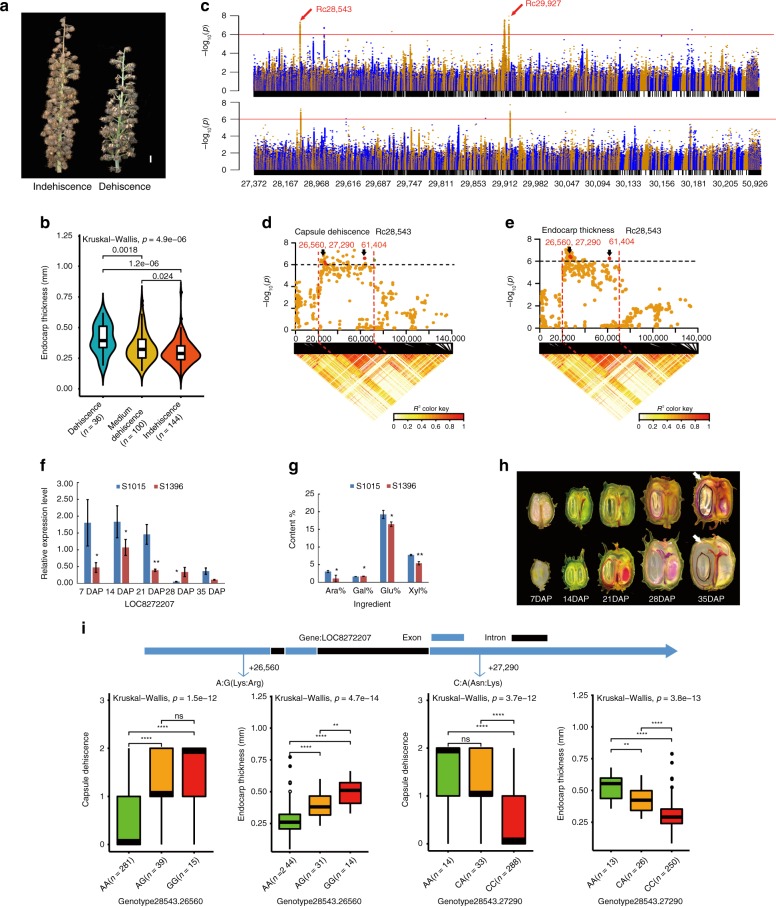
Candidate genes associated with capsule dehiscence and endocarp thickness based on genome-wide association study analysis. **a** Phenotypic difference between dehiscent and indehiscent fruit. **b** Statistical analysis of the correlation between capsule dehiscence (CD) and endocarp thickness (ET). **c** Manhattan plots for CD and ET in the full population. The horizontal red line represents the significance threshold (−log_10_*P* > 6). The arrowhead indicates the peak signal containing the candidate pleiotropic genes. **d**, **e** Local Manhattan plot (top) and linkage disequilibrium heat map (bottom) for genes associated with both CD and ET. The candidate region lies between the red dashed lines. The arrowhead indicates the three nonsynonymous single-nucleotide polymorphisms (SNPs) located in the pleiotropic gene that is co-associated with CD and ET. **f** Expression of *LOC8272207* during the development of the fruit rind in two varieties with different phenotypes, namely, S1015 (thicker endocarp and easier dehiscence) and S1396 (thinner endocarp and more difficult dehiscence), determined by quantitative reverse transcription PCR (*P < 0.05, **P < 0.01, two-tailed *t* test, three independent biological replicates). Bars donate standard deviation. **g** Analysis of pericarp composition. The cellulose and lignin contents in the fruit rinds of the S1015 and S1396 plants were measured. Ara, Gal, Glu, and Xyl are abbreviations for arabinose, galactose, glucose, and xylose, respectively. Bars donate standard deviation. **h** Lignin staining of longitudinal sections of castor fruits at different stages of development. The upper and lower five sections represent the five developmental stages of s1015 and s1396 fruits, respectively. The white arrowhead indicates the differences in endocarps. **i** The upper part shows the gene structure, and green and black rectangles indicate exons and introns, respectively; the lower box plots are for the two common nonsynonymous SNPs associated with two traits located in the candidate pleiotropic gene *LOC8282207*. The center line indicates the median; the box limits are the upper and lower quartiles, respectively; the whiskers extend to data <1.5 times the interquartile range; and the dots are outliers (**P < 0.005, ****P < 0.00005, ns indicates no significance, Kruskal–Wallis test; *n* indicates the number of accessions with the same genotype). Source data of **d**–**g** are provided as a Source Data file

Capsule dehiscence is an important yield-related trait that results in significant yield losses and harvesting difficulties. We identified the phenotypic variation in capsule dehiscence among all the castor accessions (Fig. 3a and Supplementary Fig. 1). Moreover, we observed that the percentage of dehiscence was significantly correlated with endocarp thickness (*P* < 0.01, Pearson’s product-moment correlation; Fig. 3b and Supplementary Fig. 10), with the relationship that the thicker the endocarp was, the easier the dehiscence was (*P* = 4.9e^−6^, Kruskal–Wallis test). This result implied that endocarp thickness might be a determinant of capsule dehiscence.

Using efficient mixed-model association^18^, we performed GWAS to identify potential causal genes that are significantly correlated with capsule dehiscence and endocarp thickness. Using a threshold value (−log_10_*P* > 6), we identified 171 and 48 SNPs that were significantly associated with capsule dehiscence and endocarp thickness, respectively (Supplementary Data 9, 10). By comparison, we found 5 SNPs with pleiotropic association with the dehiscence percentage and endocarp thickness in the scaffolds of Rc28543 and Rc29927 (Fig. 3c and Supplementary Fig. 11a, b). These results provide a genetic explanation for the positive correlation between dehiscence percentage and endocarp thickness. Moreover, we found that these SNPs were located in the genomic regions with a selection signal (Fig. 2d, h, i), suggesting that these SNPs could undergo positive selection for filtering castor dehiscence.

By using pairwise LD correlations, we identified 3 genes within the association signal at 20.0–70.0 kb in Rc28543 (Supplementary Data 11). Two of these 3 genes contain 3 nonsynonymous SNPs that are significantly co-associated with both the dehiscence percentage and endocarp thickness (−log_10_*P* > 6) (Fig. 3d, e). These three nonsynonymous SNPs are located in the gene *LOC8272207*, which encodes a glycosyltransferase involving the beta-1,3-xylosyltransferase pathway, and the gene *LOC8272215*, which encodes the homolog of the TRAF-like family protein in *Arabidopsis*. Quantitative reverse transcription PCR (qRT-PCR) showed that the expression of the gene encoding glycosyltransferase in the rind of fruit was significantly higher in landrace S1015 (thicker endocarp and easier dehiscence) than in landrace S1396 (thinner endocarp and more difficult dehiscence) during the development of the fruit rind at different stages (Fig. 3f). Because glycosyltransferase plays a vital role in the biosynthesis and metabolism of polysaccharide, secondary wall glucuronoxylan, cellulose, and lignin in plants^19^, we further measured the cellulose and lignin contents in the fruit rinds of S1015 and S1396 plants. We found a large difference in cellulose content and the lignin layer between S1015 and S1396 plants, showing that the thicker endocarp from landrace S1015 has more cellulose and lignin than the thinner endocarp from S1396 (Fig. 3g, h). These data suggested that the gene *LOC8272207* may be related to the accumulation of cellulose and lignin in the fruit rind and endocarp thickness. Previous studies have suggested that the accumulation of cellulose is a main reason for pod dehiscence in soybean^17,20^. Therefore, our data, coupled with the results of previous studies, suggest that capsule dehiscence of castor could be caused by the formation of dense and thick cellulose and lignin layers that sequentially increase the torsion of dried endocarp.

In addition, the gene *LOC8272215*, which encodes the homolog of TRAF-like family protein, has been shown to play a regulatory role in cell proliferation^21^. The qRT-PCR results showed that the expression of *LOC8272215* was significantly higher in landrace S1015 than in landrace S1396 during the development of the fruit rind at different stages (Supplementary Fig. 12a). Since there are no more experimental data with which to establish the link between the function of this gene and capsule dehiscence, the underlying mechanism regulating dehiscence is unclear.

By gene-based association (GBA) analysis, we found that two major haplotypes based on two nonsynonymous SNPs in the gene *LOC8272207* were significantly correlated with the dehiscence percentage and endocarp thickness. In this haplotype, SNP1 (A/G) at the 26,560 bp position in scaffold Rc28543 resulted in an amino acid change from lysine to arginine, and SNP2 (C/A) at the 27,290 bp position in scaffold Rc28543 resulted in an amino acid change from asparagine to lysine. We found that these two SNPs in the haplotype were present in the same 328 accessions, 281 of which carry the haplotype AC, which was associated with a higher dehiscence percentage and thicker endocarp, and 47 of which carry the haplotype GA, which was associated with a lower dehiscence percentage and thinner endocarp (Fig. 3i). In addition, GBA analysis revealed that the gene *LOC8272215* contains two nonsynonymous SNPs. One SNP (T/G), located at the 61,404 bp position in scaffold Rc28543, resulted in an amino acid change from glutamic acid to alanine, and the other SNP (C/A), located at the 69,981 bp position in scaffold Rc28543, caused an amino acid change from glycine to valine. The homogenous GA allele significantly increases the dehiscence percentage (Supplementary Fig. 12b).

In Rc29927, we identified one genomic region located at the position of 0–47 kb that contains 2 variants co-associated with capsule dehiscence and endocarp thickness (Supplementary Data 12). In this region, we identified one long noncoding RNA (lncRNA), *LOC108261897*, located at position 15,075–18,369bp in Rc29927. This lncRNA contains two variants (A/G and C/A) at positions 15,904 bp and 16,894 bp in Rc29927, respectively. GBA analysis also supported the association (Fig. 3c, d and Supplementary Fig. 13a, b).

Within scaffold Rc28543, we identified another gene with two nonsynonymous mutations that are associated only with capsule dehiscence. The gene *LOC8272208* also encodes a glycosyltransferase that shows 63% sequence similarity to the gene *LOC8272207*. This gene contains one SNP (A/G) located at 33,565 bp that results in amino acid changes from isoleucine to valine. The allele with A is associated with a decrease in the dehiscence percentage (supplementary Fig. 14a, b). In addition, we identified another genomic locus related to the dehiscence percentage, which is located in scaffold Rc29912. We identified seven genes containing seven nonsynonymous SNPs that were associated with the dehiscence percentage (Supplementary Data 13). The function of these genes is not well annotated, but they provide significant materials for further study.

### Candidate genes associated with hundred-grain weight

**Fig. 4:**
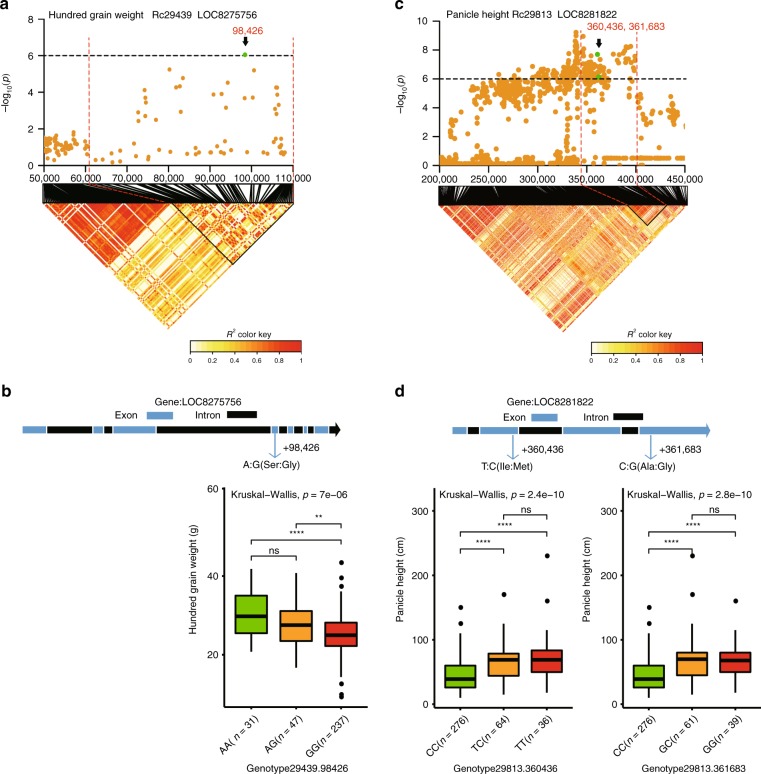
Identification of candidate genes for hundred-grain weight and panicle height based on genome-wide association study analysis. **a** Local Manhattan plot (upper) of panicle height and linkage disequilibrium (LD) heat map (lower). Green dots indicate the position of the nonsynonymous single-nucleotide polymorphisms (SNPs) located within *LOC8275756*, which was associated with hundred-grain weight. **b** The upper part shows the gene structure, and green and black rectangles indicate exons and introns, respectively; the lower box plots for the candidate gene *LOC8275756* are based on genotype. **c** Local Manhattan plot (upper) of panicle height and LD heat map (lower). Green dots indicate the position of the nonsynonymous SNP located within *LOC8281822*, which was associated with panicle height. **d** The upper part shows the gene structure, and green and black rectangles indicate exons and introns, respectively. The lower box plots for candidate gene *LOC8281822* are based on haplotypes. The center line indicates the median; the box limits are the upper and lower quartiles, respectively; the whiskers extend to data <1.5 times the interquartile range; and the dots are outliers (**P < 0.005, ****P < 0.00005, ns indicates no significance, Kruskal–Wallis test; *n* indicates the number of accessions with the same genotype)

Hundred-grain weight is an important and indispensable agronomic character in crop research that directly determines the level of production. We identified three LD blocks distributed in scaffolds Rc29439, Rc29927, and Rc29820, which contain a total of 52 significantly associated SNPs within 5 genes (Supplementary Fig. 15a, b and Supplementary Table 2). However, only one gene, *LOC8275756*, contains one nonsynonymous SNP (Supplementary Table 3). The SNP is located at 98,426 bp (A/G) in Rc29439, leading to amino acid change from serine to glycine (Fig. 4a). This gene was annotated as the homolog of the gene MEDIATOR 3 in *Arabidopsis*. Several subunits are involved in regulating plant development and metabolism, such as phenylpropanoid biosynthesis^22,23^. More importantly, it has been demonstrated that MEDIATOR 3 is involved in regulating grain size and weight in rice^22^. The accessions with the homozygous mutation genotype were significantly fewer than those with the reference genotype (Fig. 4b). According to these data, we concluded that this gene (*LOC8275756*) could be involved in regulating the grain weight of castor. In addition, for the GWAS signal in scaffold 29927, we also identified the long noncoding RNA (lncRNA) *LOC108261897*, which was significantly associated with hundred-grain weight (Supplementary Fig. 15c, d). Therefore, the lncRNA *LOC108261897* is a pleiotropic gene that is co-associated with three traits, namely, capsule dehiscence, endocarp thickness, and hundred-grain weight.

### Candidate genes associated with panicle height

Panicle height in castor is an important yield-associated trait that is correlated with the number of branches and beans. Generally, castor with shorter panicles has more branches and beans. We identified a total of three genomic loci associated with panicle height that are located on scaffolds Rc29813, Rc29912, and Rc27524 (Supplementary Fig. 16a, b). Within these three regions, we identified a total of 11 genes with nonsynonymous SNPs that were significantly related to panicle height (Supplementary Data 14). After analyzing the function of these genes, we first focused on the gene *LOC8281822*, which encodes the homolog of the r2r3-myb transcription factor LOF2 in *Arabidopsis* (Fig. 4c). LOF2 (LATERAL ORGAN FUSION2) is an MYB-domain transcription factor that is expressed in organ boundaries and functions in boundary specification, meristem initiation and maintenance, and organ patterning^24^. When the apical meristem of a plant develops into a panicle, axillary buds in the lateral organs of the stem rapidly develop into new meristems to replace it, and the cycle repeats. We thus hypothesized that this gene (LOF2) might be involved in regulating panicle height, probably by regulating axillary bud development. Furthermore, through GBA analysis, we found that this gene has two major haplotypes based on two nonsynonymous SNPs that are associated with shorter panicles (with the TG type) and taller panicles (with the CC type). In the first haplotype, one SNP (T/C) is located at position 360,436 bp in Rc29813, which results in an amino acid change from isoleucine to methionine (T/C complementary strands A/G); the other SNP (G/C) is located at 361,683 bp in Rc29813 and results in an amino acid change from alanine to glycine (G/C reverse complementary chain C/G) (Fig. 4d). We found that these two SNPs in the haplotype were present in the same 376 accessions. One hundred of these accessions, which had greater panicle height values, were associated with haplotype TG, and 276 of them, which had lower panicle height values, were associated with haplotype CC.

In addition, we identified two development-related genes, *LOC8281866* and *LOC8265107*, that were associated with panicle height. The gene *LOC8281866* encodes the homolog of cycling DOF factor 3 (CDF3), which plays multiple roles in regulating flowering time in *Arabidopsis*^25^. GBA analysis revealed two haplotypes with two nonsynonymous SNPs associated with short panicles (TG) and tall panicles (CC) in DOF3 (Supplementary Fig. 16c, d). The gene *LOC8265107* encodes the homolog of IAR3 (IAA-ALANINE RESISTANT 3) or JR3 (JASMONIC ACID RESPONSIVE 3) in *Arabidopsis*^26^. It is a member of the IAA–amino acid conjugate hydrolase subfamily and is related to the development of roots, stems, and flowers (Supplementary Fig. 16e, f).

In addition, we also performed genome-wide association analyses of five other yield-associated traits and identified tens of peak signals related to corresponding traits, including panicle length, plant height, the ratio of male to female flowers, seed length, and seed volume (Supplementary Fig. 17 and Supplementary Data 15). Among these signals, 777 SNPs were significantly associated with the corresponding traits, 26, 40, and 711 of which were located in exonic, intronic, and intergenic regions, respectively. All these findings will provide important genomic variation information for future improvement of castor characters.

## Discussion

Although a reference castor genome was released in 2010, in-depth population-based trait mapping has not been reported. Taking advantage of the Chinese collection of castor as a genetic resource, we sequenced and analyzed 405 accessions. To study the domestication history of castor in China and identify the genomic loci associated with its domestication, we first obtained 26 wild species from Africa as the control group. We show that the Chinese accessions exhibit different geographic distributions. Furthermore, the SC group appears to be the oldest Chinese population and most closely related to the wild species, whereas the MC and NC groups are clades derived from the SC group. The phylogeny suggests the possibility that the domestication or introduction of castor in China may have originated in southern China and subsequently spread into middle and northern China. Moreover, the SC and NC groups have the closest and farthest genetic distance (*F*_ST_ value) from the wild population, respectively, consistent with the phylogenetic tree. The SC group has relatively high genetic diversity (*π* value) compared with the MC and NC groups, supporting the idea that the introduction of the crop took place somewhere in southern China. However, due to the limited sampling (only 26 wild and 379 cultivated accessions) in this study, we have not yet fully uncovered the origin and detailed evolution of the Chinese castors. In addition, several phenotypes, such as yield-associated traits, changed substantially between the SC and NC groups, suggesting positive selection for local adaptation.

In the GWAS analysis, we focused on capsule dehiscence, since it is not only a key domestication target in breeding but also a trait of scientific interest. Capsule dehiscence is a key agronomical trait that was targeted by artificial selection. In soybean domestication, artificial selection for pod shattering resistance has been uncovered. Castor capsule dehiscence occurs after seed maturation, and the seed bounces out of the fruit spontaneously, which is beneficial to the reproduction and propagation of wild castor seeds in natural environments. However, this phenomenon severely restricts cultivation since it is a fatal problem that results in significant yield losses and harvesting difficulties. Therefore, indehiscence is considered as a standard for developing new industrial breeding lines. Here we propose that capsule dehiscence in castor may be caused by the formation of dense and thick lignin layers that sequentially increase the torsion of dried endocarp. The genetic variations in the gene glycosyltransferase may contribute to the accumulation of cellulose and lignin in the endocarp and thus lead to a thicker endocarp. We consider that the thicker the cellulose and lignin layers, the larger the torsion of dried endocarp, making dehiscence easier. This aspect of dehiscence could be consistent with that found in soybean and *Arabidopsis*^27,28^. The gene *Pdh1* in soybean is highly expressed in the inner sclerenchyma of soybean pod walls, which form a dense and thick fiber layer and result in pod dehiscence^20^. In *Arabidopsis*, two genes, *shp1* and *shp2*, control dehiscence zones that are lignified valve margin cells and decide whether the seed disperses^19,29^. These studies consistently suggested that the extent of cellulose and lignin could be a dominant factor affecting dehiscence.

We identified a set of candidate genes with genetic variations that were significantly associated with nine traits, namely, capsule dehiscence, endocarp thickness, hundred-grain weight, panicle height, panicle length, plant height, ratio of male to female flowers, seed length, and seed volume. The functions of some candidate genes were strongly associated with the relevant traits. However, there are some candidate genes that are uncharacterized or exhibit no direct functional link with the traits. Since a transformation system has not been created in castor, we are not able to validate the gene function by transformation in a short time. Our data provide a genetic basis for functional studies and castor breeding in the future.

## Methods

### Plant materials and genome sequencing

In the first year, 30–50 seeds of each accession were obtained from a public resource (the seed bank of oilseed plants affiliated with the Oil Crops Research Institute, Chinese Academy of Agricultural Sciences). To ensure that these seeds were not contaminated by other varieties, we planted 30 seeds of each accession to exclude samples with significant intragroup differences through field observation. To confirm that there was no serious separation among these accessions, ten seeds collected from first-year self-hybrids were planted in the ground for filtering based on experience and observation. One hybrid was selected for each accession to record all phenotypic data and for genome sequencing. Leaves at the seedling stage were collected and immediately frozen in liquid nitrogen for DNA extraction. Genomic DNA was extracted with the CTAB method^30^. According to the manufacturer’s specifications (Illumina), for each accession, high-quality DNA was required to construct a sequencing library. Paired-end sequencing (PE: 150 bp) of each library was performed on an Illumina HiSeq X-Ten system. In total, approximately 2.02 Tb of raw sequences with a 150-bp read length was generated from the Illumina HiSeq X-Ten platform.

### Phenotyping

The nine agronomic traits in our study were measured in 2017 in a field located in Wuhan, Hubei Province of China. For capsule dehiscence, we collected the lowest panicle on the main stem branches of each castor plant and scored dehiscence after air drying. Because of the asynchronization of the maturation process of capsules in the same panicle, we regarded 0–10% dehiscence of all capsules in the same panicle as indehiscence, 11–50% as slight dehiscence, and 51–100% as dehiscence; for endocarp thickness, we measured 3 replicates for each accession and obtained an average of those 3 replicates; for hundred-grain weight, we weighed 20 seeds; for panicle height, we measured from the lowest panicle to the bottom of the lowest branch on the main stem; for panicle length, we measured the length of the lowest panicle on the main stem branch; for plant height, the whole plant height was measured; for the ratio of male to female flowers, we divided the number of male flowers by that of female flowers; and for seed length, we measured the length of five seeds and obtained the average; for seed volume, because the shape of the seed is nearly an ellipsoid, we multiplied seed length by width.

### Sequence alignment and variant detection

All the sequencing reads were mapped onto the *R. communis* v0.1 reference genome on the Phytozome V10 website^1^ by the Burrows–Wheeler Aligner (v0.7.5a-r405)^31^ with default parameters. The low-quality (MQ < 20) reads were filtered in the SAMtools (v1.1)^32^ program. Picard Tools (http://broadinstitute.github.io/picard/; v1.118) was applied to coordinate, sort, and remove PCR duplicates and build a bam file index. Then the genome variants were called by Genome Analysis Toolkit (GATK)^33^. First, we performed SNP calling (GenomeAnalysisTK-3.2-2); then with conservative parameters (SNP: QD < 2.0 || MQ < 40.0 || FS > 60.0 || MQRankSum < −12.5 || ReadPosRankSum < −8.0–clusterSize 3–clusterWindowSize 10, InDel: QD < 2.0 || FS > 200.0 || ReadPosRankSum < −20.0), we filtered the first SNP calling results as known sites for base quality recalibration for the next round of SNP calling. Second, we performed a second round of SNP calling to generate GVCF files (–emitRefConfidence GVCF–variant_index_type LINEAR–variant_index_parameter 128000). Finally, all the GVCF-format files were merged for population variant calling (GenomeAnalysisTK-3.4-46) with the selected parameters (-stand_call_conf 30.0 -stand_emit_conf 40.0, SNP: QD < 2.0 || MQ < 40.0 || FS > 60.0 || MQRankSum < −12.5 || ReadPosRankSum < −8.0, InDel: QD < 2.0 || FS > 200.0 || ReadPosRankSum < −20.0).

### Variation annotation

A large number of significant SNP loci were identified based on the GWAS results. For further analysis, SNP annotation was performed according to the *R. communis* reference genome in the package ANNOVAR^34^. Based on the genome annotation, all these SNPs were categorized into exonic, intronic, intergenic, upstream or downstream regions, and splicing sites. SNPs located in coding exons were further divided into synonymous SNPs (that did not lead to amino acid changes) and nonsynonymous SNPs (that led to amino acid changes). Then the nonsynonymous SNPs were extracted for further analysis.

### Phylogenetic and population structure analysis

To identify independent SNPs for phylogenetic tree construction and structure analysis, a subset of 289,349 SNPs were extracted from the SNP data set of all accessions by applying a LD threshold of *r*^2^ < 0.05 in the program Plink (v1.90)^35^. An approximate maximum-likelihood tree was constructed with the generalized time-reversible model in the program FastTree (v2.1)^36,37^ with the formerly extracted independent SNPs. Structure analysis was performed in the program Admixture (v1.23)^38^. *K* values were set from *K* = 2 to *K* = 9. The minimum CV error value appeared at *K* = 4.

### PCA and LD decay

PCA was performed in the software GCTA (v1.26.0)^39^. The dataset for the PCA was filtered based on a quality (QUAL in the VCF file) <2000 and a scaffold length >100 kb. LD decay was analyzed by the PopLDdecay software (v3.30)^40^. A filtered (scaffold length >100 kb) VCF file was used in the analysis.

### Population genetic analysis

Fixation statistics (*F*_ST_) and nucleotide diversity (*π*) were calculated in the program VCFtools 0.1.15^41^ with a 10-kb nonoverlapping sliding window along each scaffold (length >100 kb). The nucleotide diversity and *F*_ST_ between groups were calculated based on the average value of all 10-kb sliding windows. The domestication regions of group WS vs other groups were defined by counting both the weighted *F*_ST_ value and the ratio of the *π* value in each composition window, both with a cutoff of the top 10%. The regions under selection of geographical differentiation in the domesticated groups were defined by calculating the ratio of the *π* value in each sliding window across the groups with a 5% cutoff. The scaffold order of the bar plots is in accordance with the reference genome annotation file.

### Gene Ontology

The *Arabidopsis* homologs of castor genes were searched by the blastx module of the blast program (v.ncbi-2.7.1 + )^42^. GO analysis was performed in the new version of DAVID (v6.8)^43,44^.

### GWAS analysis and identification of the candidate genes

The association analysis was performed with the Efficient Mixed-Model Association expedited (EMMAx) program^18^. The significance threshold for the associated SNPs was chosen as −log_10_*P* > 6. Several strategies were carried out to analyze the associated SNPs. First, we looked for GWAS-associated signals based on *P* < 10^−6^ and estimated the candidate regions by pairwise LD correlations. Second, on the basis of the annotation results, nonsynonymous SNPs that were significantly associated with each trait were further analyzed. Third, each of the genes containing the nonsynonymous SNPs was extracted for further functional annotation and KEGG enrichment analysis. Fourth, to analyze the function of these candidate genes more accurately, the homologs from closely related species or the model species *Arabidopsis thaliana* were identified via sequence blast.

### qRT-PCR for gene expression analysis

Five causal genes that are associated with capsule dehiscence were selected for qRT-PCR. According to the number of days after pollination (DAPs), we constructed a temporal model with five stages (every week as a stage) of the rind of castor in accessions s1015 and s1396. The seeds from plant s1015 have a thicker endocarp, and the capsule is prone to dehiscence. The seeds from plant s1396 have a thinner endocarp, and the capsule is difficult to dehisce. Total RNA was extracted with a Plant RNA Kit (BioTeke Corporation) and reverse transcribed with a PrimeScript RT Reagent Kit with gDNA Eraser (TaKaRa). qRT-PCR was performed in triplicate with SYBR Premix Dimer Eraser (Perfect Real Time) (TaKaRa). Gene expression levels were calculated with the comparative −ΔΔCt method^45^. Primers were designed by Primer3 (http://bioinfo.ut.ee/primer3-0.4.0/). Their sequences are listed in Supplementary Table 4.

### Lignin-stained slices

The seeds were harvested at different developmental stages according to the DAP and stored in FAA fixative (containing 45% ethanol, 6% acetic acid, and 5% formaldehyde) before use. The seeds were then sliced lengthwise along the back suture by hand sectioning, and the slices were spread across a glass slide. Then 40% hydrochloric acid was added dropwise onto the slices to cover their surface and allowed to rest for approximately 10 min. The excess liquid was siphoned off, and the slices were stained with 5% phloroglucinol for approximately half an hour until the lignin was stained completely.

### Reporting summary

Further information on research design is available in the Nature Research Reporting Summary linked to this article.

## Supplementary information

Supplementary Info

Peer Review

Reporting Summary

Description of Additional Supplementary Files

Supplementary Data 1

Supplementary Data 2

Supplementary Data 3

Supplementary Data 4

Supplementary Data 5

Supplementary Data 6

Supplementary Data 7

Supplementary Data 8

Supplementary Data 9

Supplementary Data 10

Supplementary Data 11

Supplementary Data 12

Supplementary Data 13

Supplementary Data 14

Supplementary Data 15

Source Data

## Data Availability

Data supporting the findings of this work are available within the paper and its Supplementary Information files. A reporting summary for this article is available as a Supplementary Information file. The datasets generated and analyzed during the current study are available from the corresponding author upon request. Sequencing data that support the findings of this study have been deposited in Sequence Read Archive (SRA), NCBI with the accession code PRJNA548999. Source data underlying Figs. 1c, d, f and 3d–g as well as Supplementary Figs. 2, 4b, 6, and 12a are provided as a Source Data file. SNPs and InDel data can be downloaded from ftp://ftp.agis.org.cn/~cuipeng/castor-VA/.
