# Supplementary material for: Sequencing of Chinese castor lines reveals genetic signatures of selection and yield-associated loci
Source: Nat Commun. 2019 Jul 31;10:3418. doi: 10.1038/s41467-019-11228-3 (PMC6668449; doi:10.1038/s41467-019-11228-3)
Supplement: Supplementary file 1 — Supplementary Info [file 41467_2019_11228_MOESM1_ESM.pdf]

Sequencing of Chinese castor lines reveals genetic signatures of selection and yield-associated loci

Fan *et al.*

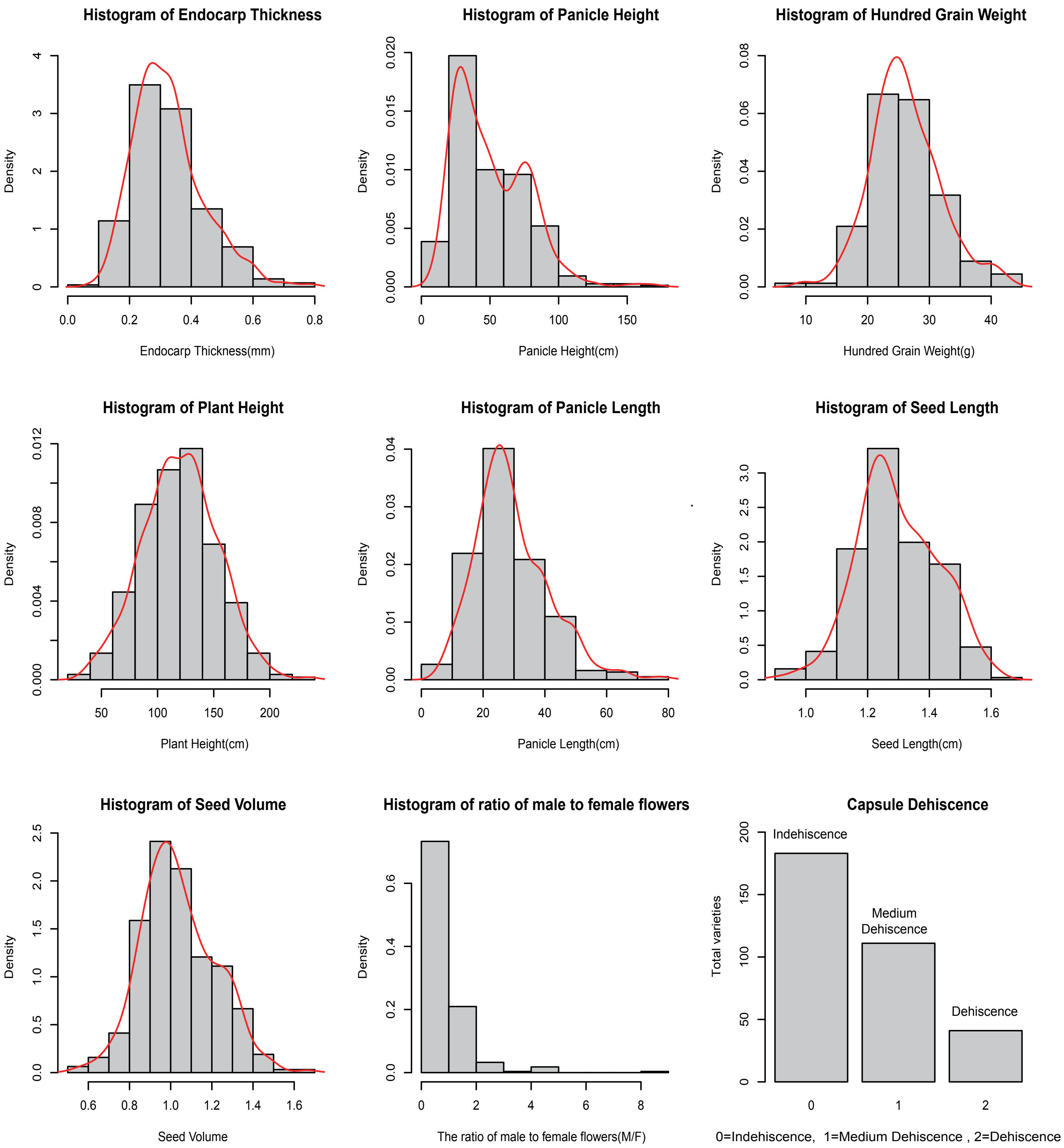

Supplementary Figure 1 Histogram of nine agricultural traits.

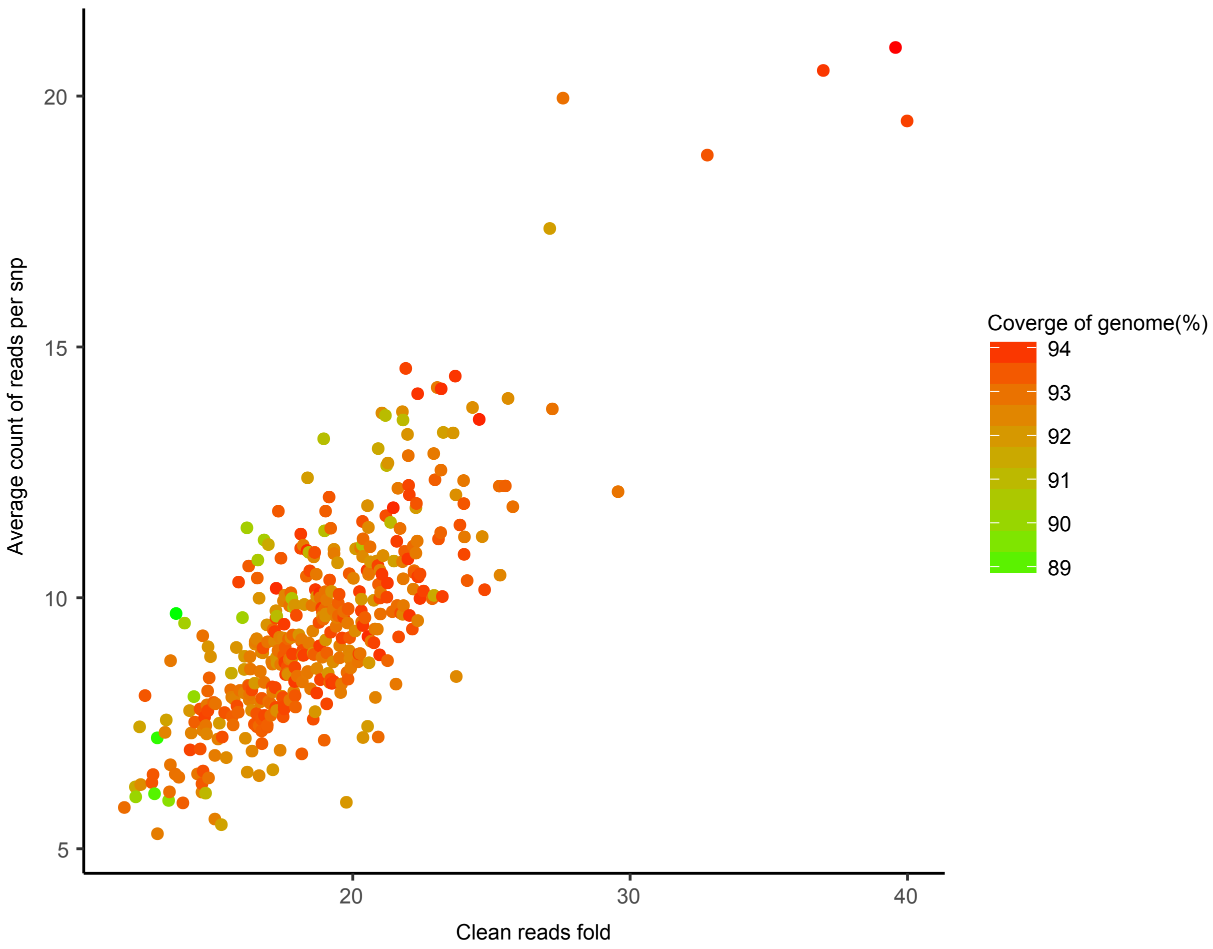

Supplementary Figure 2 Depth and coverage distribution of all accessions. x-axis: Sequencing depth distribution after cleaning filtration (total bases number of clean reads/genome size), y-axis: average count of reads per snp in each accession , color: genome coverage ratio of sequenced data after cleaning filtration. Source data of Supplementary Figure 2 are provided as a Source Data file.

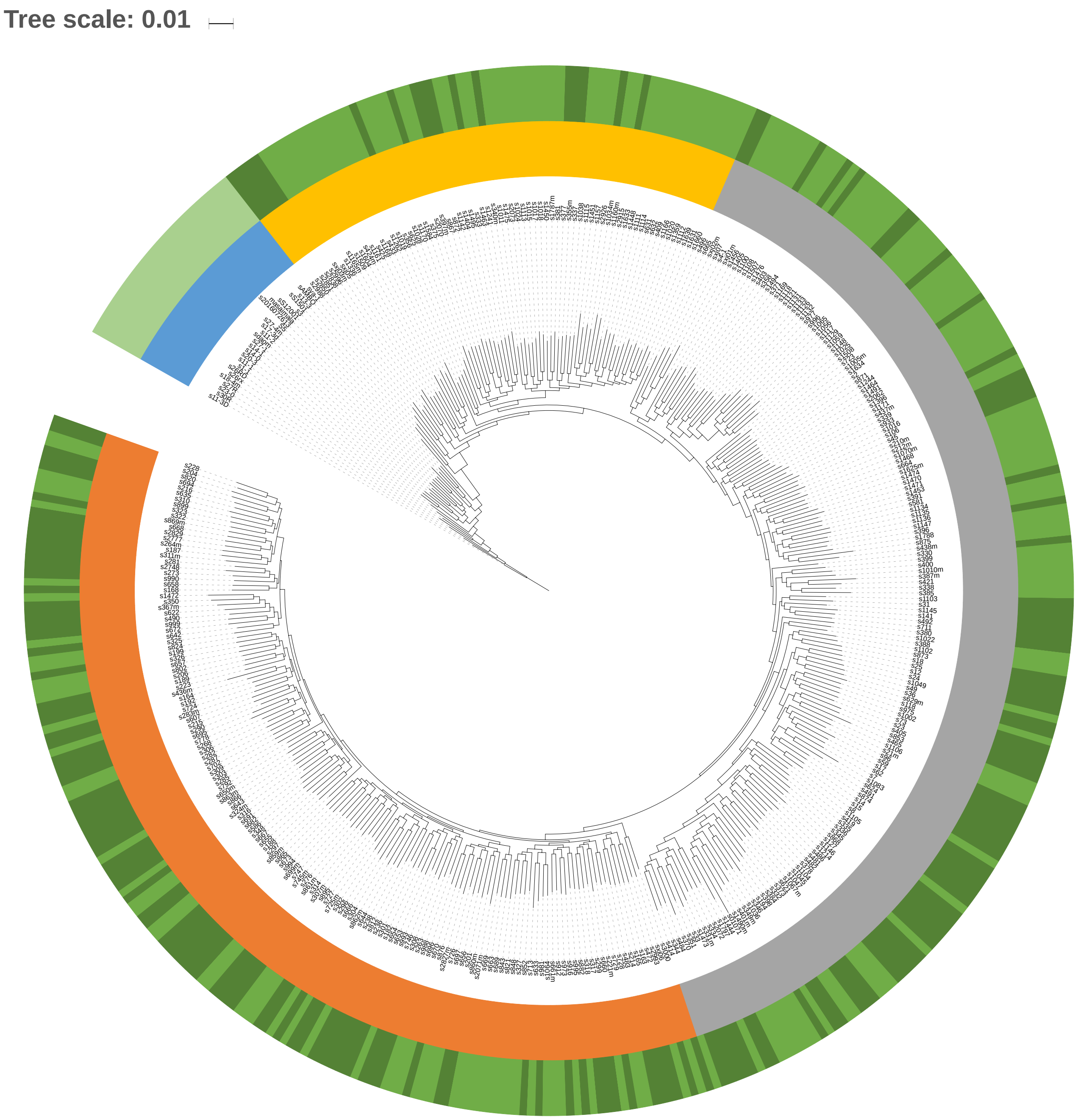

Supplementary Figure 3 Phylogenetic tree with branch length and accessions annotation. Inner color strip: group by phylogenetic tree. blue: group WS; yellow: group SC; grey: group MC; orange: group NC. Outer color strip: group by sample domestication infomation on record. light green: group wild species; medium green: landrace; dark green: breeding line.

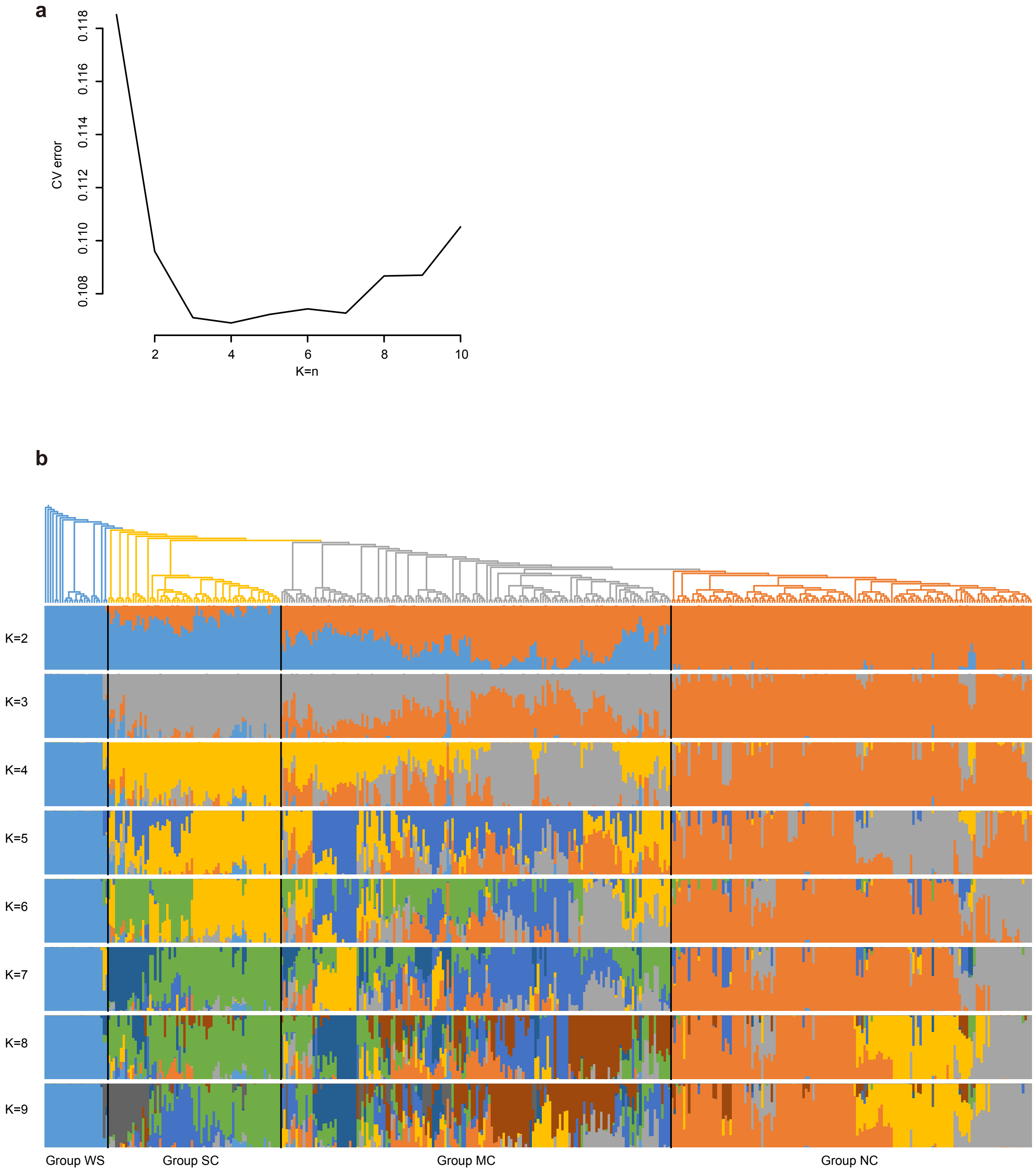

Supplementary Figure 4 Structure analysis of all accessions. (a) CV error line chart from K=1~10, minimum value appeared in K=4 which represents the optimum group number. (b) Structure bar plot from K=1 to K=9, match to the phylogenetic tree and group, color: ingredient in each accessions. Source data of Supplementary Figure 4b are provided as a Source Data file.

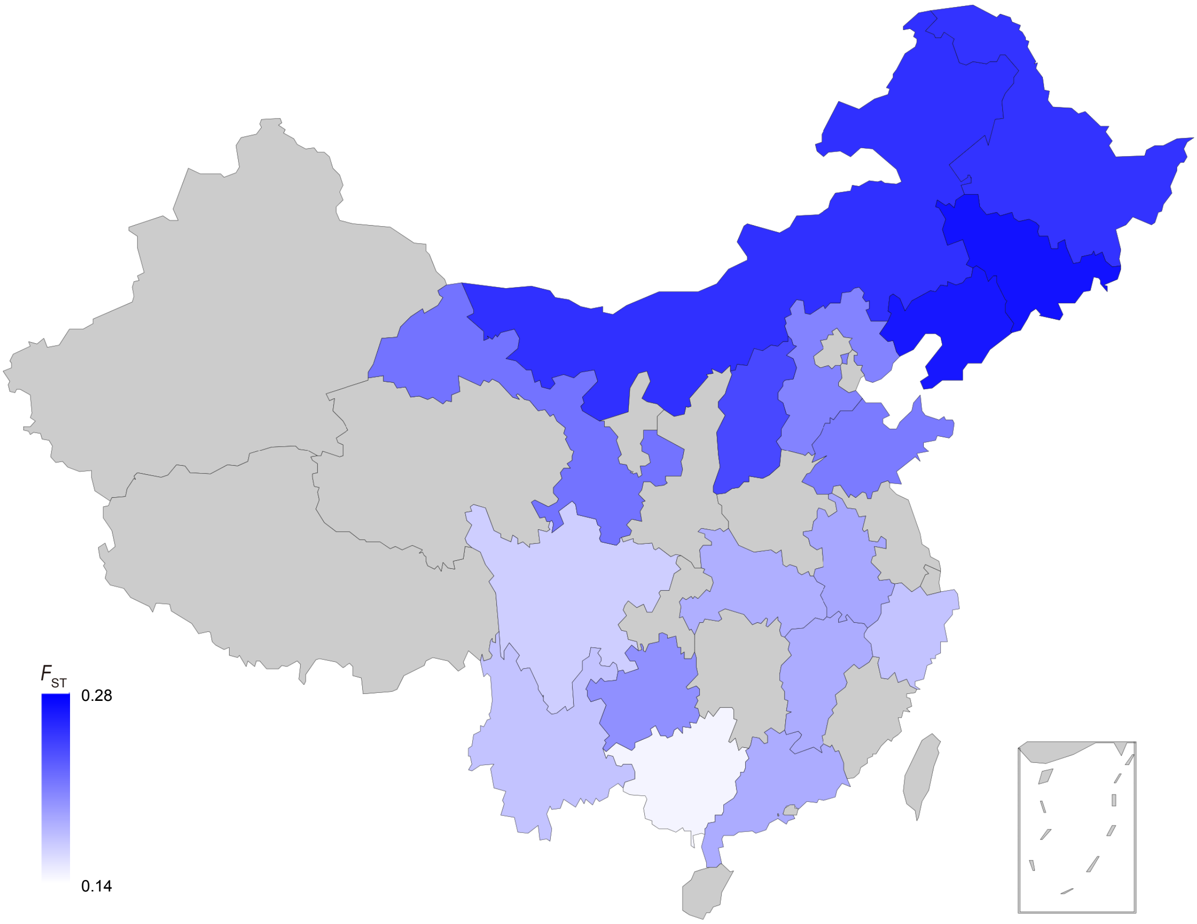

Supplementary Figure 5 Group WS vs. each province of China F_{ST} value distribution. Each province in the figure has more than three samples. Blue depth is according to the mean F_{ST} value of each province, grey means the number of sample less than three.

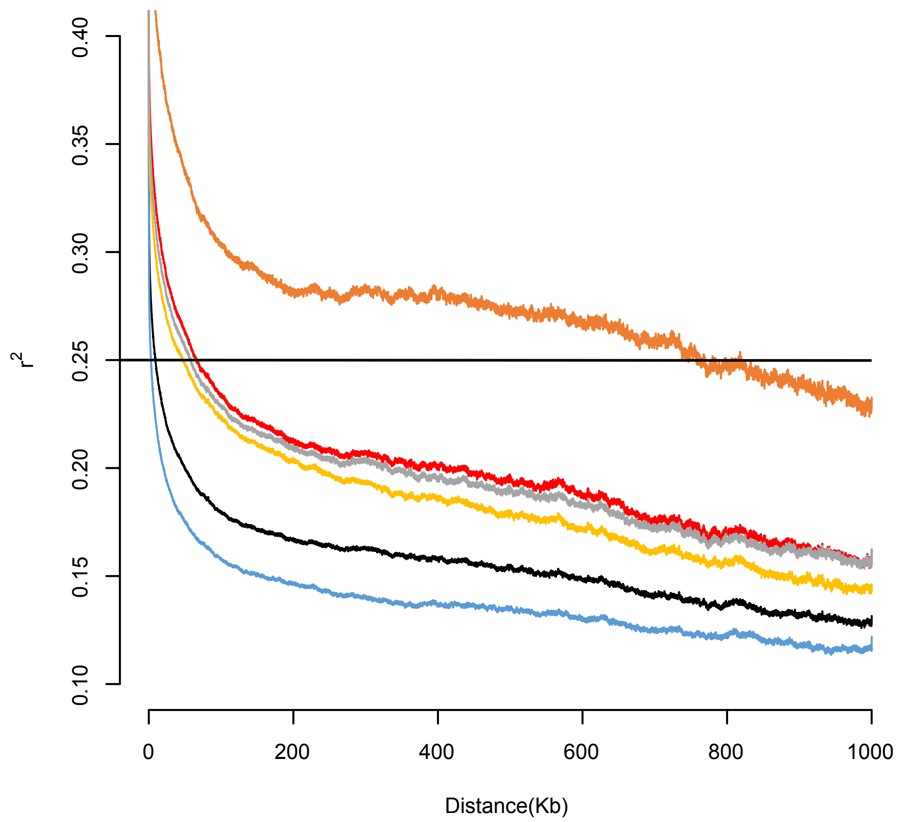

Supplementary Figure 6 Linkage disequilibrium decay of different groups. black: all accessions; blue: Group WS red: all except Group WS; yellow: Group SC; grey: Group MC; orange: Group NC; line: r^2=0.25 as a stantard of comparation between different groups. Source data of Supplementary Figure 6 are provided as a Source Data file.

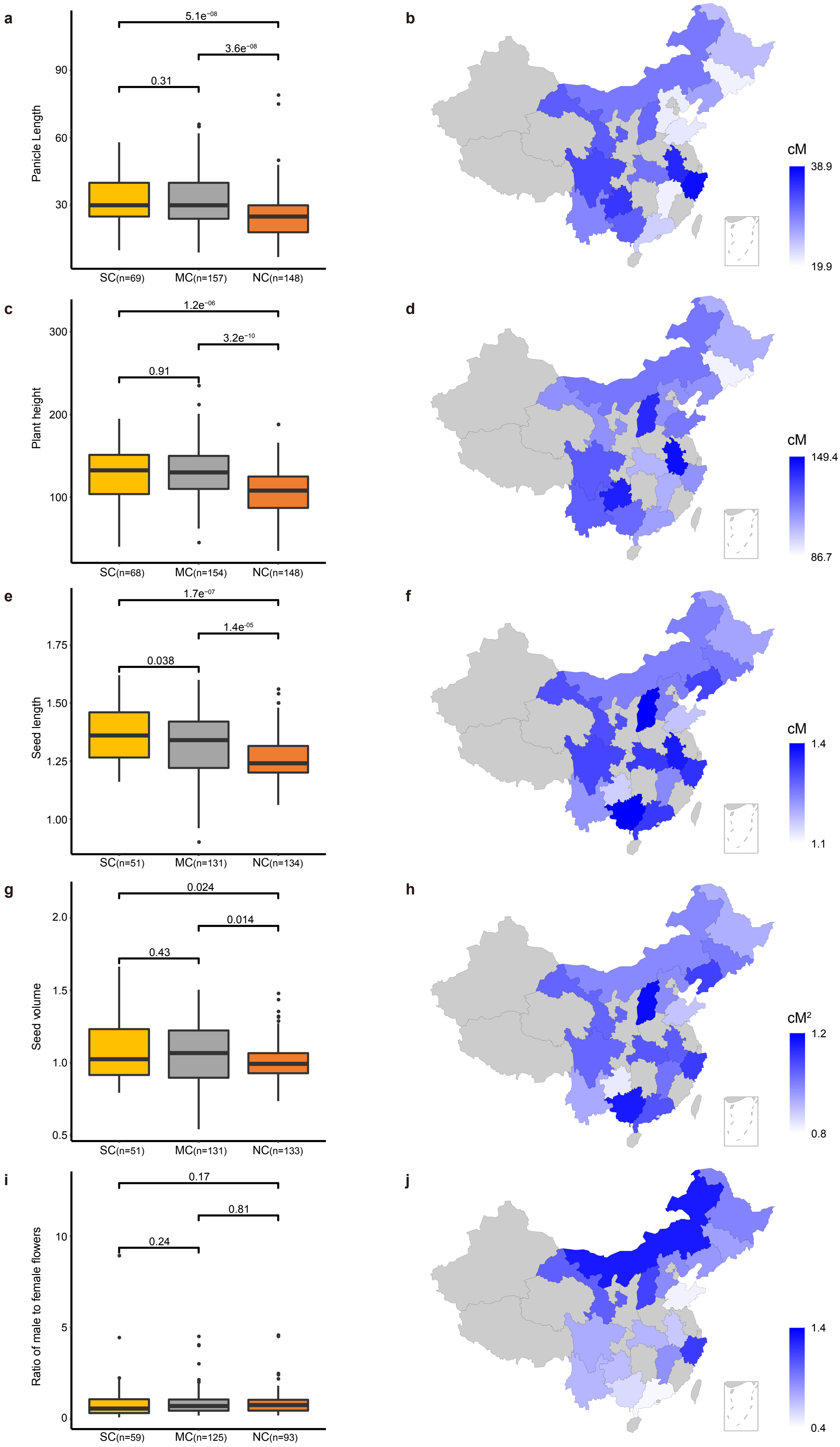

Supplementary Figure 7 Five phenotypes intergroup statistic and geographical distribution in China. (a) (c) (e) (g) (i): boxplot of phenotype in each group with *p*-value showed, yellow:group SC; grey: group MC; orange: group NC. Center line marks median. Box limits are upper and lower quartiles. Whiskers extend to data less than 1.5 times the interquartile range. Dots represent outliers. (p-value showed, wilcoxon test.) (b) (d) (f) (h) (j): heatmap of phenotype in each province in china, each province in grey means there has no sample or no more than 3 samples from the district.

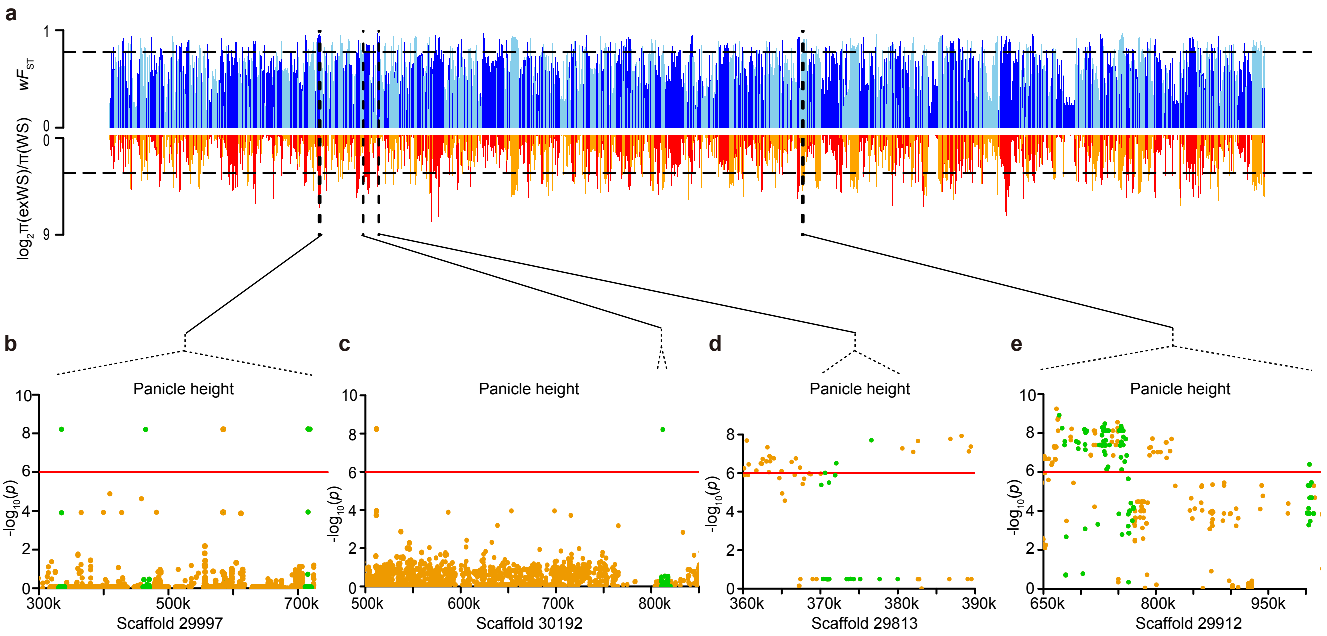

Supplementary Figure 8 Domestication region of Group WS vs. All other groups except Group WS(Group exWS) merge to partial GWAS results. (a): wF_{ST} value and π ratio with 10% top line of Group WS vs. Group exWS (b)-(e): GWAS sites which merge to the domesticaion region.

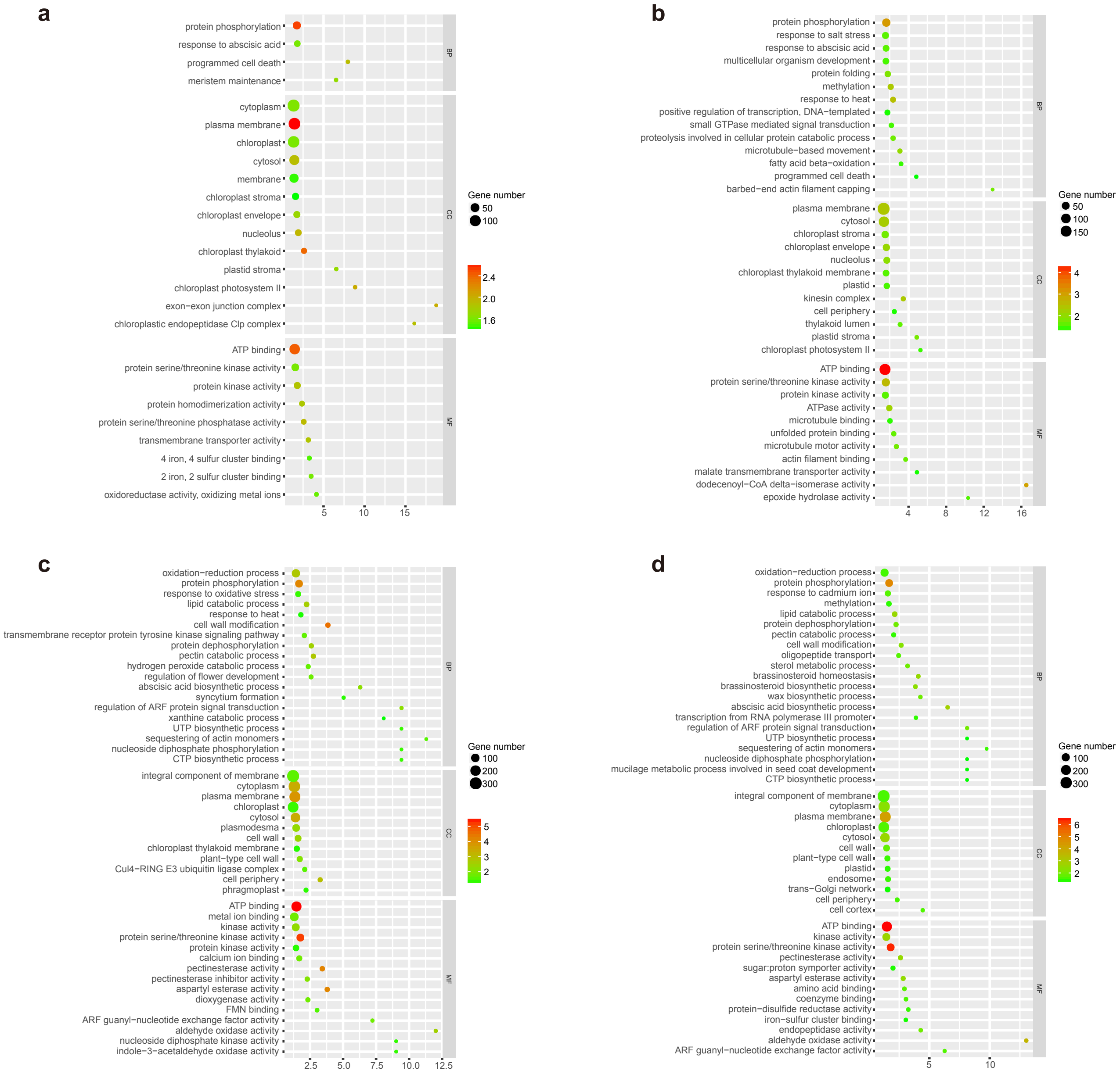

Supplementary Figure 9 Go analysis of domestication genes blast to arabidopsis thaliana. x-axis: folds enrichment; y-axis: pathway name; color: $-\log_{10}$(*p*-value), *p*-value: modified Fisher's exact *p*-value. (a):Group WS vs. All groups except Group WS ; (b): Group SC vs. GroupMC; (c): Group MC vs. GroupNC; (d): Group SC vs. GroupNC.

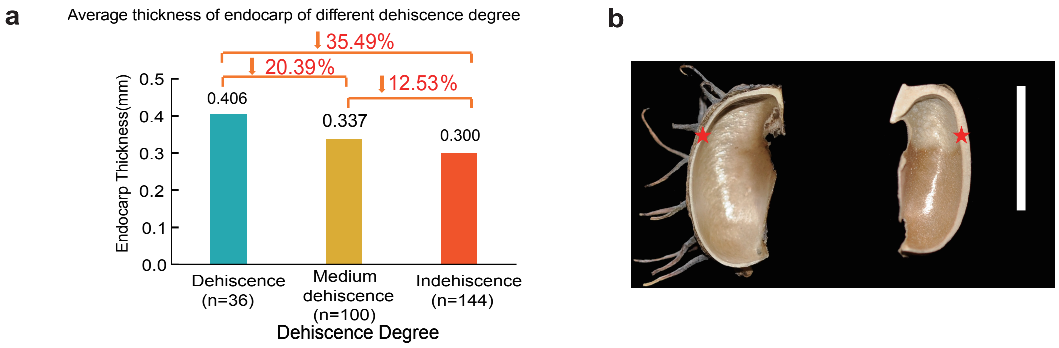

Supplementary Figure 10 Statistical analysis of the correlation between capsule dehiscence and endocarp thickness. (a)Average endocarp thickness of three different dehiscence degree populations which were divided into based on the dehiscent percentage. (b)The difference of endocarp thickness between dehiscent and indehiscent landraces. Red asterisks mark the position of endocarp where we measured.

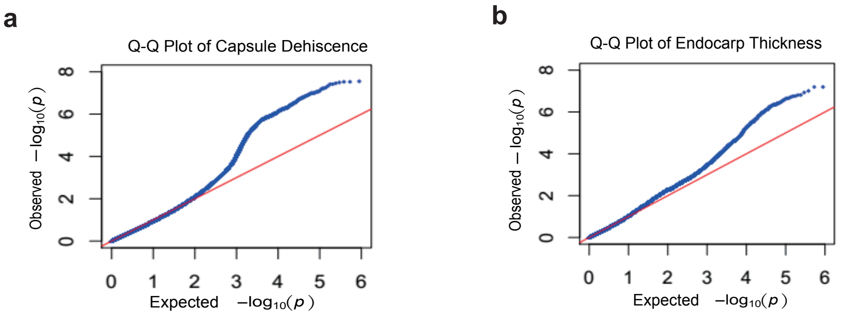

Supplementary Figure 11 Quantile-quantile plots of observed versus expected $-\log_{10}$ (P) of GWAS results. The red line in each plot represents an idealized case where theoretical test statistic quantiles match simulated test statistic quantiles. (a) Capsule Dehiscence. (b) Endocarp thickness.

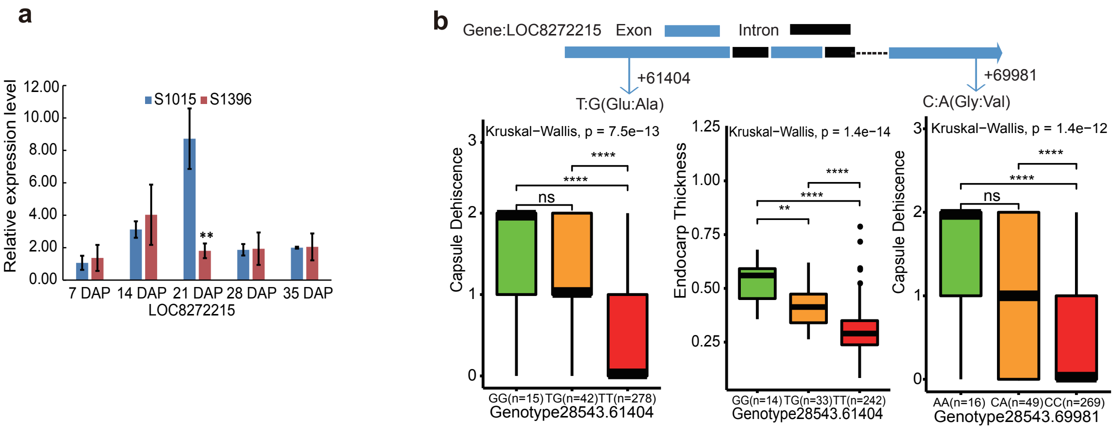

Supplementary Figure 12 Comparison of gene *LOC8272215* expression and box plots for haplotypes. (a)Comparison of gene *LOC8272215* expression in the developing process of the fruit rind of two different phynotype varieties S1015 (thicker endocarp and easier dehiscent) and S1396 (thinner endocarp and harder dehiscent) by qRT-PCR (***P*<0.01,two-tailed t-test,three independent biological replications). Bars donate standard deviation. (b)The upper shows gene structure,green and black rectangles indicate exons and introns, respectively. The lower box plots for capsule dehiscence and endocarp thickness for haplotypes. Center line marks median. Box limits are upper and lower quartiles.Whiskers extend to data less than 1.5 times the interquartile range.Dots represent outliers (***P* < 0.005,*****P* < 0.00005,ns represents no significant,Kruskal-Wallis test.The more the asterisks, the more significant the difference). Source data of Supplementary Figure 12a are provided as a Source Data file.

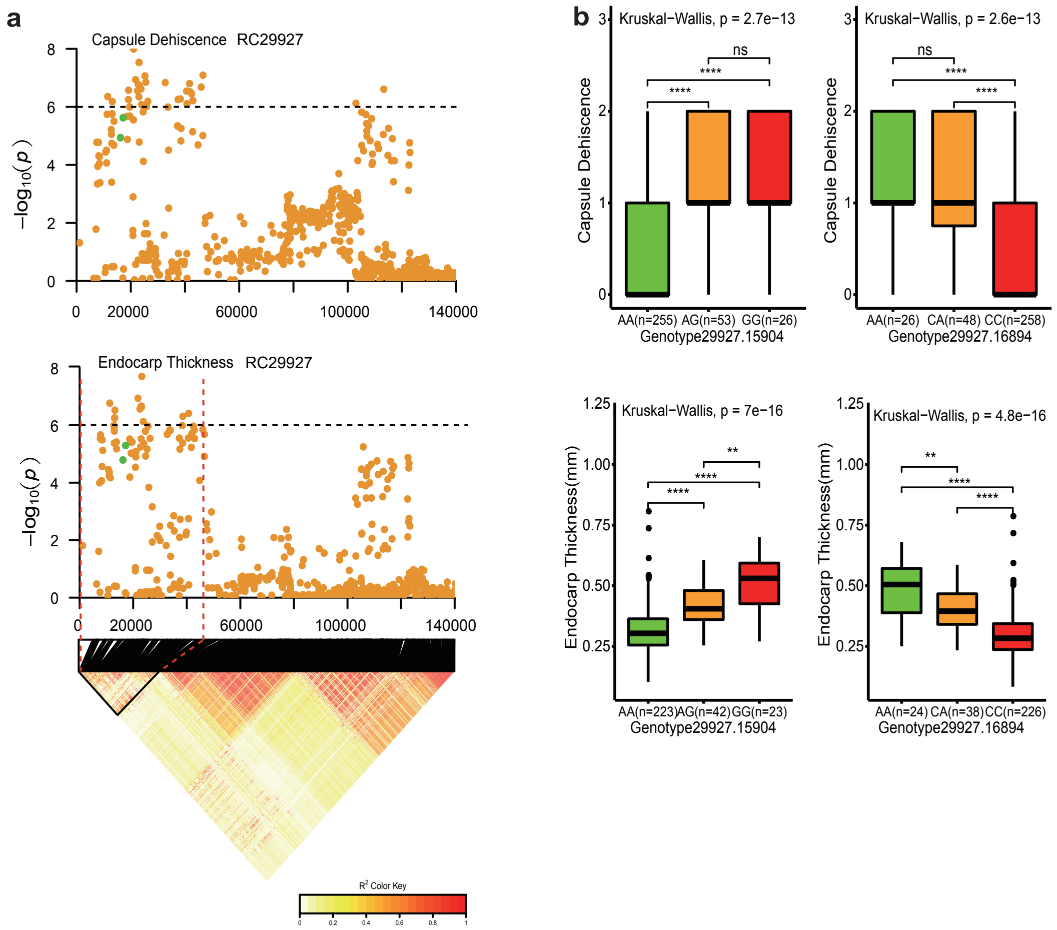

Supplementary Figure 13 GWAS for capsule dehiscence and endocarp thickness and identification of the pleiotropic gene *LOC108261897*. (a)Local Manhattan plot(upper) for the two traits capsule dehiscence and endocarp thickness and LD heat map(lower). Horizontal black dashed line represents the significance threshold -$\log_{10}(P)$>6.Green dots indicate the position of common nucleotide variation in the candidate gene. The red dashed lines indicate the candidate region. (b)The upper box plots show the haplotypes for capsule dehiscence. The lower box plots for endocarp thickness. Center line marks median. Box limits are upper and lower quartiles. Whiskers extend to data less than 1.5 times the interquartile range. Dots represent outliers (**P < 0.005,****P < 0.00005, ns represents no significance, Kruskal-Wallis test). The more the asterisks, the more significant the difference).

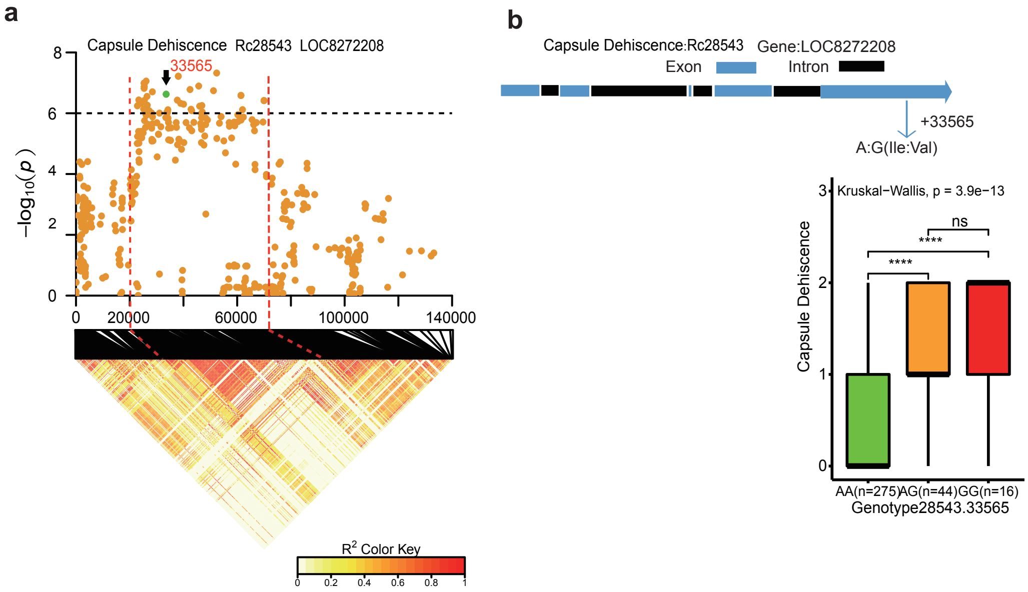

Supplementary Figure 14 GWAS for capsule dehiscence and identification of the causal gene *LOC8272208*. (a)Local manhattan plots(upper) for capsule dehiscence and LD heat map(lower). Horizontal black dashed line represents the significance threshold (–log10 (*P*)>6). Green dots indicate the position of nonsynonymous SNP which was located within *LOC8272208*. Double red vertical dashed lines indicate the candidate region between 22.6 and 70.0 kb (b) The upper shows gene structure, green and black rectangles indicate exons and introns, respectively. The lower box plots for candicate gene *LOC8272208* based on haplotype. Center line marks median. Box limits are upper and lower quartiles. Whiskers extend to data less than 1.5 times the interquartile range. Dots represent outliers (*****P* < 0.00005, ns represents no significant,Kruskal-Wallis test. The more the asterisks, the more significant the difference).

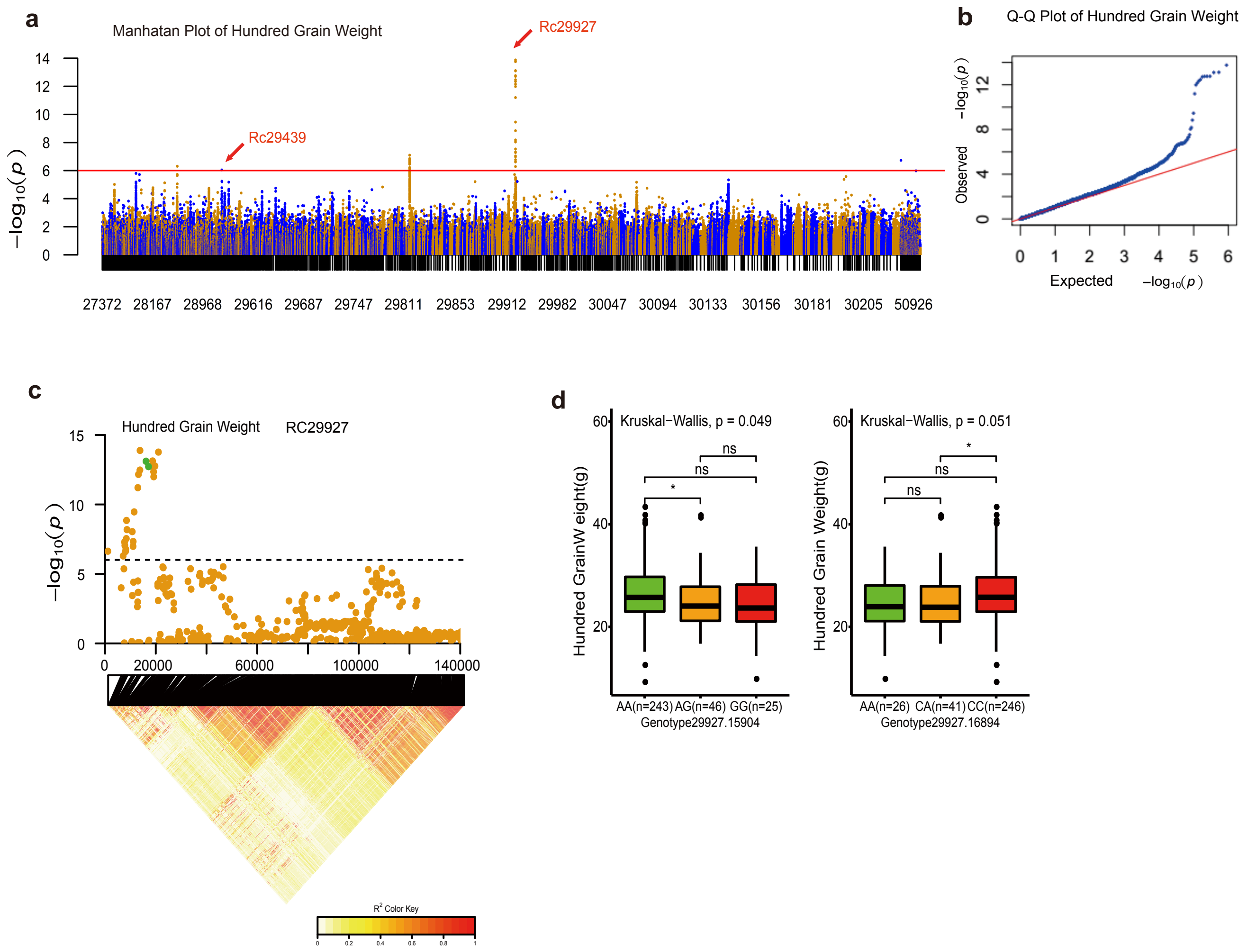

Supplementary Figure 15 GWAS for hundred grain weight and identification of the causal genes. (a)Manhattan plots for hundred grain weight.Horizontal red line represents the significance threshold -log₁₀(*P*)>6.The arrowhead indicates the peak signal containing the candidate genes. (b) Quantile-quantile plots. (c)Local Manhattan plot(upper) of panicle height and LD heat map(lower) .Horizontal black dashed line represents the significance threshold $-\log_{10}(P)>6$.Green dots indicate the position of two SNPs which were located within a pleiotropic gene *LOC108261897*.(d)Box plots for candicate gene *LOC108261897* based on haplotype.Center line marks median.Box limits are upper and lower quartiles. Whiskers extend to data less than 1.5 times the interquartile range.Dots represent outliers (**P* < 0.05 , ns represents no significant, Kruskal-Wallis test. The more the asterisks, the more significant the difference).

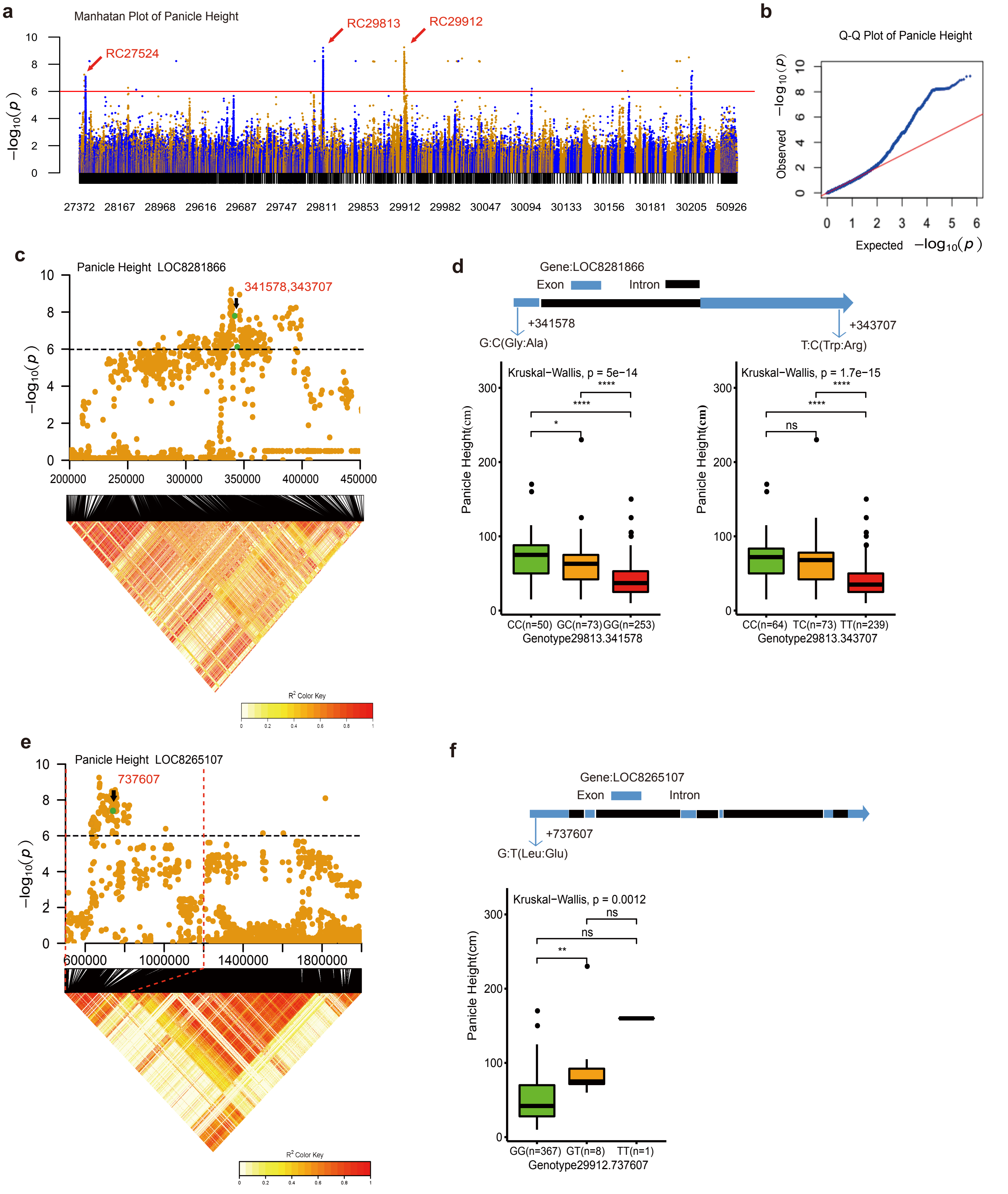

Supplementary Figure 16 GWAS for panicle height and identification of the causal genes. (a)Manhattan plots for panicle height. Horizontal red line represents the significance threshold (-$\log_{10}P$>6).The arrowhead indicates the peak signal containing the candidate genes. (b)Quantile-quantile plots. (c)Local Manhattan plot(upper) of panicle height and LD heat map(lower). Green dots indicate the position of two nonsynonymous SNPs which were located within *LOC8281866*. (d)The upper show gene structure,green and black rectangle indicate exons and introns, respectively.The lower box plots for candicate gene *LOC8281866* based on haplotype. (e)Local Manhattan plot (upper) of panicle height and LD heat map (lower). Red dots indicate the position of nonsynonymous SNP which was located within *LOC8265107*. (f)The upper show gene structure, green and black rectangle indicate exons and introns, respectively.The lower box plots for candicate gene *LOC8265107* based on haplotype.Center line mark median. Box limits are upper and lower quartiles. Whiskers extend to data less than 1.5 times the interquartile range.Dots represent outliers (* $P < 0.05$, ** $P < 0.005$, **** $P < 0.00005$, ns represents no significant, Kruskal-Wallis test. n indicates the number of accessions with the same genotype. The more the asterisks, the more significant the difference).

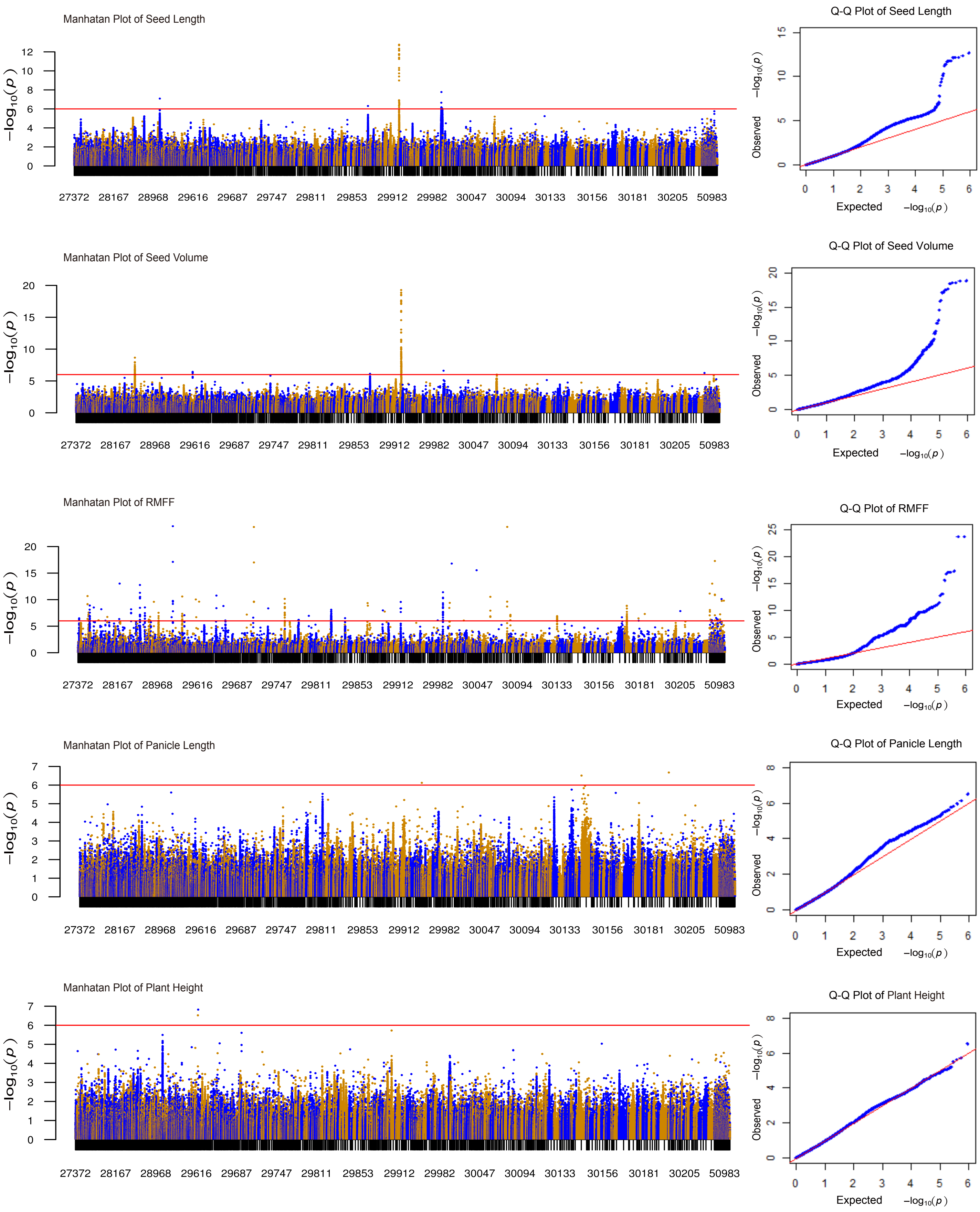

Supplementary Figure 17 Manhattan and Q-Q plots of five agronomic traits of castor bean. On the left are Manhattan plots of seed length, seed volume, RMFF, panicle length and plant height from top to bottom; On the right is the corresponding Q-Q plots. Horizontal red line represents the significance threshold $-\log_{10}(P)>6$. RMFF indicates the ratio of male to female flowers.

Supplementary Table 1 SNPs classification

SNP function		SNPs counting
UTR3		6400
UTR5		3848
upstream		208682
upstream;downstream		24785
downstream		178780
intergenic		3245321
splicing		705
intronic		249714
exonic	stopgain	1907
	unknown	388
	stoploss	274
	synonymous SNV	57393
	nonsynonymous SNV	79523

Supplementary Table 2 Statistic SNP(-log10P＞6) significantly associated with hundred grain weight

Scaffold	Count	Exonic region	Exon SNPs number	Exon SNPs variation type	Gene name
Rc28543	1	intergenic	-	-	-
Rc29439	1	exonic	1	nonsynonymous SNV	29439.t000013
Rc29820	1	exonic	1	synonymous SNV	29820.t000006
Rc29820	20	intergenic	-	-	-
Rc29820	2	upstream	-	-	29820.t000006
Rc29927	26	intergenic	-	-	-
Rc30752	1	intergenic	-	-	-
Total	52	-	2	-	-

Supplementary Table 3 Annotations of nonsynonymous SNV associated with hundred grain weight

Scaffold	Position	*p* -value	Ref	Alt	Annotype
Rc29439	98426	9.04E-07	A	G	nonsynonymous SNV

Amino acid Change	RC.anno	pacId	locus name	transcript name
29439.m000227.TIGRR0.1:exon4:c.A973G:p.S325G	conserved hypothetical protein	16802869	29439.t000013	29439.m000227

Peptide name	NCBI Anno	Pfam	Panther
29439.m000227	mediator of RNA polymerase II transcription subunit 27 (LOC8275756), mRNA	PF11571	PTHR13130,PTHR13130:SF4

KO	Best-hit-arabi-name	Best-hit-gene name
K15170	AT3G09180.1	LOC8275756

Supplementary Table 4 Primers for qRT-PCR

Gene name	Best-hit gene name	Primer-F	Primer-R
28543.t000003	LOC8272207	TACGAAGTAGCTAGCCCTAGAA	GGCTCCAGTCAAGAACATCA
28543.t000008	LOC8272215	CAAGTGCAAGGACAGAACCA	TCAGAGCTGTTCATGCAAGG
28543.t000010	LOC8272215	CATATTCCCAACCCGAAATG	TGTGAACTTTGTGGGGTTCA
RcActin		AGTCTTGTTCCAGCCATCTCTC	CAGTGATCTCCTTGCTCATACG

Supplementary Table 5 Primers for SNPs verification

Scaffold	Target start	Target end	Primer-F	Primer-R	F_start	F_end	R_start	R_end
27524	51090	51790	AGAGAGAGGTTGACTAAAGCATTCA	GCCATCCTGTGAATTCCCTGTT	51004	51028	51856	51835
29719	171364	172064	TGGCTAGTAGATGTGTGATTTGC	TCCAAGATTAGGGCTTGCATCT	171321	171343	172100	172079
29719	198625	199325	CGTGAGATTGAGAAGTGAAAAAGTG	TGCTTTCAGCATTTAGCAATCC	198538	198562	199424	199403
29813	259522	260222	AGATGGTGAAAGCTACGTGGG	AGGTAGAAGTAAATGTGGTGTACT	259476	259496	260281	260258
29835	133333	134033	TGCCTTTCACAGCCGTAAGT	TGCCAATTCATCTCTTGAGTTTGAC	133253	133272	134085	134061
29912	640877	641577	AAAGATTGGTTGTGCTGTGTGT	AGAACCCTACCCCATCCCAG	640833	640854	641664	641645
29912	685376	686076	CATGTGCACCCGAAACTTCC	GAAACGATGACCCGCAATTCT	685335	685354	686124	686104
29912	731665	732365	TATGCAGGATTCTCAGCCCC	TCTGGATAAAAGAAATTGGATCGG	731608	731627	732394	732371
29912	753225	753925	TTTTGCTTCCTTTTTGAATTGTGTT	CCCTCCTACGTGTTTGATTGC	753170	753194	754004	753984
29912	1432963	1433663	TGTGCTCAAGCAATAGATGCAG	GCGTCCCAACACACGAAGTA	1432915	1432936	1433695	1433676
29912	1450326	1451026	TCACAAGTACAGAGCGGCTT	ACTGTCATCGTCGACTTCTTCA	1450250	1450269	1451098	1451077
29912	1458795	1459495	GAGGCCAATGTCTCCTGGAA	ACATCAGTACTTCCCGGCAT	1458717	1458736	1459580	1459561
29912	1497561	1498261	GAGGGAACGAATTGCTCTTCA	TACCCAAAGACACCCACTCTC	1497532	1497552	1498307	1498287
29912	1548895	1549595	TCAGTTTTGGCCTTGAAATGCTT	CCTGTTGTGGACCACTCAGAT	1548798	1548820	1549636	1549616
29912	1627366	1628066	TTCCTTTTGTTGATCAGGGATTGC	GTGGCCATATGTTCAGGTTAATTG	1627278	1627301	1628092	1628069
29912	1657594	1658294	AGGCCTTTTTGGGTACTTGTCA	CTCCAGACGCAGGATGGTTT	1657495	1657516	1658341	1658322
29989	216870	217570	TGAGAATCAAAATGGACACTCACC	TGGCGAGTTGTTAGAGACTGT	216845	216868	217661	217641
30133	140255	140955	TGGAGAGAGTATTTCGAAGGAGTT	ATTCTTGTGCGTGTCAGGCT	140176	140199	141007	140988
