# Supplementary material for: Sequencing of Chinese castor lines reveals genetic signatures of selection and yield-associated loci
Source: Nat Commun. 2019 Jul 31;10:3418. doi: 10.1038/s41467-019-11228-3 (PMC6668449; doi:10.1038/s41467-019-11228-3)
Supplement: Supplementary file 4 — Description of Additional Supplementary Files [file 41467_2019_11228_MOESM4_ESM.docx]

Description of Additional Supplementary Files

File Name: Supplementary Data 1
Description: List of Sample collection and information

File Name: Supplementary Data 2
Description: Sequencing data, alignment and SNPs status

File Name: Supplementary Data 3
Description: Castor groups divided by phylogenetic tree

File Name: Supplementary Data 4
Description: The genomic regions associated with geographic differentiation

File Name: Supplementary Data 5
Description: The genomic regions associated with both geographic differentiation and GWAS

File Name: Supplementary Data 6
Description: The genomic regions associated with domestication

File Name: Supplementary Data 7
Description: The genomic regions associated with both domestication and GWAS

File Name: Supplementary Data 8
Description: GO analysis for genes associated with domestication

File Name: Supplementary Data 9
Description: Total SNPs significantly associated with capsule dehiscence (-log10P>6)

File Name: Supplementary Data 10
Description: Total SNPs significantly associated with endocarp thick (-log10P>6)

File Name: Supplementary Data 11
Description: LD block RC28543 for capsule dehiscence(20.0-70.0kb)

File Name: Supplementary Data 12
Description: LD block RC29927 for capsule dehiscence(0-47.0kb)

File Name: Supplementary Data 13
Description: LD block RC29912 for capsule dehiscence (1237.4-1663.6kb)

File Name: Supplementary Data 14
Description: List of all associated genes for Panicle height

File Name: Supplementary Data 15
Description: Total significantly associated SNPs corresponding to five yield-related traits
