# Supplementary material for: Sequencing of Chinese castor lines reveals genetic signatures of selection and yield-associated loci
Source: Nat Commun. 2019 Jul 31;10:3418. doi: 10.1038/s41467-019-11228-3 (PMC6668449; doi:10.1038/s41467-019-11228-3)
Supplement: Supplementary file 2 — Peer Review [file 41467_2019_11228_MOESM2_ESM.pdf]

Reviewers' comments:

Reviewer #1 (Remarks to the Author):

The authors present a genetic analysis of a number of traits in a GWAS population of castor bean. The population consists of a smaller group of lines which are effectively defined as 'ancestor' populations, through their origin in Africa, compared to material currently grown within China. The SNO analysis for the material is done through resequencing of a population of 405 lines and a marker set developed to investigate genetic structure, diversity, potential selective sweeps in cultivated material (African vs China; Pi) and to evaluate a number of traits by GWAS. The phenotypic dataset was collected through the growth of the lines over two years in Wu Han. Average values over two years were used (?) based on 10 plants per line, although experimental design for the trial is not mentioned (RCBD, line-rows etc) and there is no independent statistical test presented for the trial data, such as ANOVA. If the line data is being combined across years, it would also be good to see how consistent it is per year. Figure 1 supplemental presents the distribution of the traits and a fitted line, but not formal analysis of whether the distributions are normal or how strong the genetic effect is, based on an ANOVA that relates directly to the experimental design. Both would be useful. In addition, there is a special focus on seed capsule cracking as a specific trait that does directly effect yield (recovery).
As the authors do present whisker and box plots for the traits, there has been some trait analysis, but it would be good if this could be more clearly examined in the paper, as it underpins their title and GWAS data.
The majority of the approaches adopted by the authors appear appropriate and well used elsewhere. The only potential question would be whether the (small) African group adequately represents the 'wild' material, although Pi values for this are higher than for the geographically separated groups (as determined by STRUCTURE and PCA analysis), although are lower than the comparisons to largely inbreeding species and the rapid drop in LD suggests an outbreeding history. Fst values compared to the wild material do suggest that Southern China was probably the site of introduction, with subsequent spread northwards.
The 9 traits analysed from the breeding material gave rise to a wide range of GWAS signal. The traits are correctly labelled as 'yield-associated', rather than yield traits and comparison the nucleotide diversity seen in cultivated material compared to 'wild' material identified a number of location where there may have been selection acting during introduction to China or subsequent selection/breeding. Seed capsule cracking was specifically investigated (presumably because it is a reasonably understood biomechanical trait in other beans (and especially soybean) where the physical cause (increased thickness and lignification of the capsule) is known and QRT-PCR was used to investigate gene expression levels of 5 candidate genes.

For a number of traits, potential candidate genes are identified and discussed.

Overall, the manuscript is well written (although there are mistakes in English which need to be corrected) and clear. The claims in the discussion are likely to be justified, assuming that their point that this is the first time such an analysis has been carried out in this species it also true.
While there is nothing revolutionary in the applied methods (and more detail on phenotypes is needed), the authors present a well-structured and comprehensive analysis of breeding material within China which will be useful for marker-assisted selection and improvement of agricultural traits in this species

Reviewer #2 (Remarks to the Author):

This manuscript was well written reporting sequence of some Chinese castor lines, identification of nearly three hundred thousand SNPs, determination of the population structure and genetic diversity of Chinese castor germplasm, and association of some candidate genes with useful traits. The authors

analyzed the data properly and made some important conclusions. The originality or uniqueness of this manuscript was the authors first time did some gene-based association analysis with yield-related traits such as seed weight, panicle size, plant height, and capsule dehiscence in castor. The information presented in the manuscript is very useful for castor geneticists and breeders in the castor field, and also for other scientists. However, I think there are several weakness for this manuscript. To further improve the quality of this manuscript, I have the following concerns, suggestions, and comments. Please see attached file in details.

Reviewer #3 (Remarks to the Author):

I have gone through the MS and found it impressive in terms of number of accessions they have sequenced. Castor bean is a crop of high economic importance. This manuscript reports the sequencing of 405 cultivated and wild castor bean accessions with a goal of identification of genomic loci associated with selection and yield. The study reports several candidate genes associated with agronomic traits like capsule cracking, endocarp thickness, ear height, seed volume. The study can be a useful resource for genetic studies in castor bean and for breeding community.

However, I feel the authors did not justify the huge data generated in the study. Along with SNPs, the authors could have looked into structural variations such as copy number and presence/absence variations as structural variations are also reported to have significant associations with phenotypic variations.

Some other important points are listed below:

1.The methodology of SNP calling and filtering is not clear. The GATK version should be specified in the MS.

2. The readability of the MS needs to be considerably improved. For instance, Lines 211, 214, 521 look incomplete.

3. The authors should refine the language and also check for typological errors such as Chinses in Line 152, peal at Line 412.

4.The authors should use more technical and acceptable terms for data. For instance, for average genome depth, instead of folds, X can be used. I believe the authors mean "calculating genomic windows" instead of "genomic calculating windows".

5. In line 526, the length should be kb instead of k.

6. While mentioning about genomic regions, the authors should state the scaffold number followed by coordinates instead of first coordinates and then scaffold number. In Line 525: The dataset for the PCA analysis was filtered with the quality less than 2000; I understand the authors are talking about VCF file but I am not sure which particular quality variable was filtered by 2000.

7. References should be cross checked. Reference 31 is incorrectly cited at its second instance and the author name should be Heng Li instead of Heng L. GCTA tool for PCA anlaysis should be correctly cited. The format of references is not uniform in the MS.

Dear editors and reviewers,

Thanks a lot for raising the valuable questions and suggestions for our manuscript "Resequencing 405 cultivated and wild castor bean accessions identified genetic signature of selection and yield-associated loci", which is indeed helpful for improving our manuscript. We have revised the manuscript according to your kind advices and suggestions one by one.

Response to Peer Review Comments

Reviewer #1

COMMENT 1: The authors present a genetic analysis of a number of traits in a GWAS population of castor bean. The population consists of a smaller group of lines which are effectively defined as 'ancestor' populations, through their origin in Africa, compared to material currently grown within China. The SNO analysis for the material is done through resequencing of a population of 405 lines and a marker set developed to investigate genetic structure, diversity, potential selective sweeps in cultivated material (African vs China; Pi) and to evaluate a number of traits by GWAS. The phenotypic dataset was collected through the growth of the lines over two years in Wu Han. Average values over two years were used (?) based on 10 plants per line, although experimental design for the trial is not mentioned (RCBD, line-rows etc) and there is no independent statistical test presented for the trial data, such as ANOVA. If the line data is being combined across years, it would also be good to see how consistent it is per year. **RESPONSE:** This is a very good comment. We realized that this part content in our manuscript was not stated precisely, which lead to be misunderstood. In fact, we selected 10 plants for each accession that were used for getting the plants with stable phenotype; however, we did not record all phenotypic data for all 10 plants. At this stage, we only depended on experience and observation, which aim to confirm that samples do not exist serious separation situations among those accessions. We selected one of ten plants for recording all phenotypic data, and then the selected one was just used for sequencing. Because we considered that each measured phenotype data should have an exactly corresponding genome to be

sequenced for genome wide association analysis. In a short summary, reviewer's suggestion is very good, but we did not record the phenotypic data from all 10 plants from each accession due to the shortcoming of labor. Instead, in order to filter out the random error and environmental effects on plant growth and phenotypic measurement, we tied to used more accessions (379 in total) that were sequenced and used for GWAS analysis. We considered that there should be little possibility to get a GWAS correlation by a random error.

We only recorded the second-year phenotypic data, and did not record the first-year data. We here would like to explain the concrete process of sampling. At the first year, we obtained about 30~50 seeds for each accession from one public resource (the seed bank of oilseed plant affiliated to oil crops research institute, Chinese Academy of Agricultural Sciences). However, at this stage, we were not sure if these seeds are purified or these seeds might be mixed by other kinds of castor seeds. And thus, we planted 30 seeds for each accession in the same condition and filtered out the plants that show significant differences compared with others among the grown 30 plants according to the morphological features such as plant height, leaf shape, leaf margin, stem branching, flowering time. After filtering, the plants, that are consistent with each other at the phenotype, were self-hybridized to generate the homozygotes. In summary, for the first year, we tried to get the purified seeds and did not record the data. At the second year, we planted 10 seeds generated by self-hybridization for each accession. If all 10 plants appear to be consistent at the morphological features, we would pick one of ten plant for phenotypic data collection and sequencing. Otherwise, we gave up the accession that did not show consistent phenotype. Based on the reviewer's comment, we should and will keep recording the phenotypic data in the following years. We will integrate these data in the future, and cannot add these data into the current manuscript version. However, we added the details for sampling process in the method part (page 13, line 527-543) and stated more clearly this time.

Additionally, we added the details that how each phenotype was measured (page 13, line 546-561). For capsule dehiscence, we collected the lowest panicle in main stem

branch of each castor, and counted dehiscence after dry by airing simultaneously. Because of the asynchronization maturing process of capsules in same panicle, we regarded 0-10% dehiscence of all capsules in same panicle as indehiscence, 11-50% as slight dehiscence, 51-100% as dehiscence, respectively; for endocarp thickness, we measured three times for each accession and got the average of 3 times; for hundred seed weight, we weighed 20 seeds and got the average value; for panicle height, we measured the lowest panicle from ground to the bottom in main stem branch; for panicle length, we measured the length of the lowest panicle in main stem branch; for plant height, the whole plant height were measured; for the ratio of male and female flowers, we divided the number of male flowers by female flowers; for seed length, we measured the length of five seeds and got the average; for seed volume, because the shape of seed is nearly a ellipsoid, we regard value that multiply seed length by width as volume.

COMMENT 2: Figure 1 supplemental presents the distribution of the traits and a fitted line, but not formal analysis of whether the distributions are normal or how strong the genetic effect is, based on an ANOVA that relates directly to the experimental design.

RESPONSE: I understand what is this comment meaning. Here, we only aim to illustrate the enough variations of the phenotypes, which is fit for performing the genome-wide association analysis. Due to lacking of phenotypic data, we were not able to do more analysis to show how strong the genetic or environmental effect. Thanks for this suggestion and it is really a good comment.

COMMENT 3: The only potential question would be whether the (small) African group adequately represents the 'wild' material.

RESPONSE: This is a concern. To be honest, we are not very clear about the status of wild population of castor beans in Africa, presently. 26 samples may be inappropriate to represent all wild species of castor. Due to the limit of oversea sampling, only 26 wild species from African were obtained and used for this analysis. However, it is very sure that all these 26 species are wild castors because they grow at

the wilds and show distinct at the morphology from the cultivated caster. Additionally, as reviewer see, the pi and LD decay in these 26 samples are consistent with the expectation that one wild population should have. Therefore, we consider that it should not be a big affect on the conclusion that we got. However, to keep scientific, we added some discussion to describe this concern in the manuscript (page 12-13, line 482-484)

COMMENT 4: The traits are correctly labelled as 'yield-associated', rather than yield traits

RESPONSE: Surely, that expression is more precisely, and we have changed in the manuscript of new version.

COMMENT 5: there are mistakes in English which need to be corrected.

RESPONSE: Mistakes that we found in manuscript have been corrected in the new version.

Reviewer #2

Major revisions:

COMMENT 1: Terminology issues: There are several terminology words which have to be replaced with the right ones throughout the text. Capsule crack: the right botanical word should be capsule dehiscence. Ear length: Corn has ear but not castor. The right botanical word should be panicle length. Seed long: It should be seed length. Castor bean: Castor does not belong to the legume. The accurate name for it should be castor (see Severino et al. 2012).

RESPONSE: Thanks for your comment. We already revised them according to your suggestion.

COMMENT 2: Title of the manuscript: The authors did not sequence these 405 Chinese castor lines before. They used resequencing. It may refer to Chan et al. 2010 paper. Other word should be used to replace resequencing. Castor bean was also used

improperly. In addition, castor lines used were mainly from China. In the manuscript title, this fact should be reflected. Therefore, the manuscript title should be revised.

RESPONSE: According this suggestion, we would like to revise tile "Resequencing 405 cultivated and wild castor bean accessions identified genetic signature of selection and yield-associated loci" **to "Sequencing Chinese Castor lines Identified Genetic Signatures of Selection and Yield-associated Loci"**

COMMENT 3: Castor line selection: Experimental materials are very important for the conclusion. The authors did not mention why and how these 405 lines/accessions were selected for this research. They should give the reason.

RESPONSE: This comment is reasonable. We selected 405 samples based on three-point considerations. First, to study the domestication history of castor in China and identify the genomic loci associated with domestication, we firstly obtained 26 wild species from Africa as the control group. Due to the limit of oversea sampling, only 26 samples were obtained. Second, to study the population structure of Chinese castor lines and their geographical differentiation, we tried to collect samples extensively that is across China from Southern to Northern, and totally get 379 samples from 26 provinces that cover almost all the castor-cultivation regions. Third, considering the GWAS analysis, we selected the samples that have a enough variations of phenotype among the accessions. These contents have added into result part in the new version (page 3, line 123-131)

#COMMENT 4: Dehiscence: Dehiscence is an important trait for several crops (such as sesame). However, the authors did not mention how the dehiscence affect the harvesting time and yield in the introduction and discussion. After reading the manuscript, I did not have a hint whether dehiscence is an ideal trait or not in castor. What is the breeding object for dehiscence in castor? The breeders should select dehiscence trait. Which type? 0, 1, or 2 type? The authors should address this question in the discussion.

RESPONSE: According to this comment, we added several sentences to address the importance of capsule dehiscence in the discussion(page 12，line 490-498). "capsule

dehiscence is a key agronomical trait, which was targeted by human selection. For example, in soybean domestication, artificial selection for pod shattering resistance has been uncovered. Castor capsule dehiscence occurs after seed maturation and the seed bounces out of the fruit spontaneously, which is beneficial to the reproduction and propagation of wild castor seeds in natural environment. However, it severely restricts cultivation, since it is the fatal problem that results in significant yield losses and harvesting difficulties. Therefore, indehiscence has already been considered as a standard for developing new industrial breeding lines."

COMMENT 5: Linkage groups: Knowing the sequence or candidate genes on chromosome locations or linkage groups is very important. I wonder whether the authors have linkage information for the sequence they generated. If so, they should provide to readers or deposit the information in the database.

RESPONSE: This is a very good suggestion. However, the information about linkage group is not available in castor**.**

COMMENT 6: Compare population structure and genetic diversity with other castor studies. Reports on castor population structure and genetic diversity are available. The authors should compare their results with others.

RESPONSE: This is a good suggestion. Actually, several months ago, we already noticed two relatively comprehensive papers that published by the same group: one is *Castor Bean Organelle Genome Sequencing and Worldwide Genetic Diversity Analysis* (Maximo R, *PLoS ONE*, 2011); the other one is *Single nucleotide polymorphisms for assessing genetic diversity in castor bean (Ricinus communis)* (Foster J T, *BMC Plant Biology*, 2010). In these two articles they reported the evolution of castor all over the world, via genotyping the chloroplast from 894 accessions and 48 genome loci (SNPs) from 488 germplasm samples. Actually, we would like to investigate the relationship between Chinese lines and worldwide species through integrating our data and their data. However, unfortunately we can not access the data from them. We have sent E-mail to the research group and asked for the raw data that was not submitted with the paper; however, the group have not

yet provided the data to us so far.

#COMMENT 7: Complementation test by transformation: To finally confirm the candidate gene for the traits investigated, the complementation test has to be performed. The authors did not do this experiment. I suggest that the authors should mention it in the discussion.
RESPONSE: This is a good reminding. Since the transformation system can not be created in castor presently, we can not validate the gene function by transformation in a short time. We mention this point in the discussion part (page 12, and line 519-521).

COMMENT 8: Add methods used for collection of plant traits in supplementary materials: The authors did not give detailed information on the methods they used for collection of plant traits (capsule dehiscence, endocarp thickness, panicle length, panicle height, seed length, seed volume, and others). They should add a single file for it in the supplementary materials.
RESPONSE: This is a good reminding. We added a part content to tell how we measured all the phenotypes in the manuscript (page 13, line 546-561) and that can also be seen in the following. "The nine agronomic traits in our study were measured in 2017 at the field located at Wu Han province of China. For capsule dehiscence, we collected the lowest panicle in main stem branches of each castor, and counted dehiscence after dry by airing simultaneously. Because of the asynchronization of maturing process of capsules in same panicle, we regarded 0-10% dehiscence of all capsules in same panicle as indehiscence, 11-50% as slight dehiscence, 51-100% as dehiscence, respectively; for endocarp thickness, we measured 3 times for each accession and got the average of 3 times; for hundred grain weight, we weighed 20 seeds to evaluate the value; for panicle height, we measured the lowest panicle from ground to the bottom of lowest branch from main stem; for panicle length, we measured the length of the lowest panicle in main stem branch; for plant height, the whole plant height were measured; for the ratio of male and female flowers, we divided the number of male flowers by female flowers; for seed length, we measured the length of five seeds and got the average; for seed volume, because the shape of

seed is nearly a ellipsoid, we regard value that multiply seed length by width as volume. ”

Minor revisions:

COMMENT :

1. Line 314, “gene base association” should be “gene based association”.
2. Line 373, 374, 395-396, I do not understand the meaning of higher ear and lower ear. Please rewrite these sentences.
3. Line 412, “peal” or “real”?
4. Line 465, what did you mean “Ten filled seeds”? “Young leaves were collected from where? At which stage?
5. Line 474-485, how many plants are you measured for these agronomic traits? 10 plants?
6. Line 555, “Arabidopsis thaliana” should be in italics.
7. Line 572, “developing times” should be “developing stages”.

RESPONSE:

1. Mistake were corrected in the new version.
2. We rewrote these sentences.
3. We corrected it.
4. It is truly a ambiguity word, which originally means plump seeds , and “young” was also inaccurate we substituted it for “at seedling stage”.
5. One plant was used for the measurement.
6. Mistake was corrected in the new version.
7. Mistake was corrected in the new version.

Reference format:

Keep consistent for the journal name abbreviation and format. If you use abbreviation for the journal names, you have to use abbreviation for all journal names. For reference 1, you used Nat Biotechnol but for reference 2, you used Journal of Ethnopharmacology.

RESPONSE: Thank you for pointing out the mistake, which was revised in the new version manuscript.

Reviewer #3

COMMENT 1': did not justify the huge data generated in the study. Along with SNPs, the authors could have looked into structural variations such as copy number and presence/absence variations as structural variations are also reported to have significant associations with phenotypic variations.

RESPONSE: These are good questions. To justify data, we tested 98 SNP sites by applying PCR and sanger sequencing. 99.61% of the SNPs in the test can be detected by sanger sequencing. This result suggested the high reliability for SNP identification. This result was added into the new version manuscript. (page 4, line 148-152)

According to the comment, we tried to call CNVs in each sample using the software callCNVfree and related them to the phenotype. Firstly, Long scaffold(>=100 kilo bases) was selected as a reference for CNV calling. Each scaffold was split into10 kb window with step 2 kb. In each window, reads coverage were calculated and adjusted by GC-content. To find if there is any CNV related to phenotypes, GWAS analysis was also performed by applying software Plink (v1.0.7 because new version remove the CNV-related functions). The result show that there is no CNV reaching the cutoff line($p<10^{-6}$), which can be seen by an example in the following figure This part of analysis suggested that there might be no significant correlation between CNVs and phenotypes.

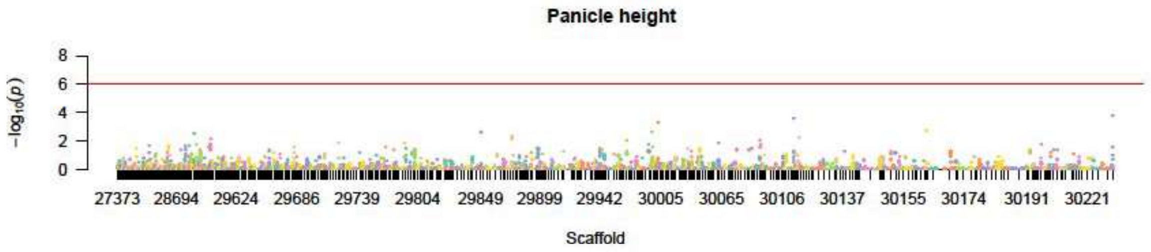

COMMENT 1: The methodology of SNP calling and filtering is not clear. The GATK version should be specified in the MS.

RESPONSE: We illustrated the methodology of SNP calling and filtering in method part in the new version manuscript (page 14, line 564-582). Also see here "After BWA

alignment. The low quality (MQ<20) reads were filtered in sam tools (v1.1). Picard-tools (v1.118) was applied to sort, remove PCR duplicates and build bam file index. Then, the genome variants were called by Genome Analysis Toolkit (GATK). There are three main steps. Firstly, We performed first SNP calling(GenomeAnalysisTK-3.2-2), and then through harsh parameter(SNP: QD < 2.0 || MQ < 40.0 || FS > 60.0 || MQRankSum < -12.5 || ReadPosRankSum < -8.0 --clusterSize 3 --clusterWindowSize 10, InDel: QD < 2.0 || FS >200.0 || ReadPosRankSum < -20.0) of filter we filtered first SNP result as a known site training data for the next round SNP calling. Secondly, we preformed next round SNP calling to generate GVCF files (--emitRefConfidence GVCF --variant_index_type LINEAR --variant_index_parameter 128000). At last, all the GVCF format files are merged for population variants calling(GenomeAnalysisTK-3.4-46) with parameters (-stand_call_conf 30.0 -stand_emit_conf 40.0 SNP: QD < 2.0 || MQ < 40.0 || FS > 60.0 || MQRankSum < -12.5 || ReadPosRankSum < -8.0, InDel: QD < 2.0 || FS >200.0 || ReadPosRankSum < -20.0).”

COMMENT 2: The readability of the MS needs to be considerably improved. For instance, Lines 211, 214, 521 look incomplete.

RESPONSE: Thanks for checking the readability of MS. Mistakes were corrected in the new version (page 6, line 231-233; page 6, line 233-235; page 14, line 601-602;).

#COMMENT 3: The authors should refine the language and also check for typological errors such as Chinses in Line 152, peal at Line 412.

RESPONSE: Manuscript has been revised in new version.

#COMMENT 4: The authors should use more technical and acceptable terms for data. For instance, for average genome depth, instead of folds, X can be used. I believe the authors mean "calculating genomic windows" instead of "genomic calculating windows".

RESPONSE: Sure, the symbol “×” is widely used in genomic researches. Disorder of phrases have been revised in new version.

#COMMENT 5: In line 526, the length should be kb instead of k.

RESPONSE: Mistakes were corrected in the new version of MS.

#COMMENT 6: While mentioning about genomic regions, the authors should state the scaffold number followed by coordinates instead of first coordinates and then scaffold number. In Line 525: The dataset for the PCA analysis was filtered with the quality less than 2000; I understand the authors are talking about VCF file but I am not sure which particular quality variable was filtered by 2000.

RESPONSE: We have changed the order of scaffold number and coordinates in the manuscript of new version. Quality filtering was for depth control, so we used QUAL value in VCF file as a marker of depth.

#COMMENT 7: References should be cross checked. Reference 31 is incorrectly cited at its second instance and the author name should be Heng Li instead of Heng L. GCTA tool for PCA analysis should be correctly cited. The format of references is not uniform in the MS.

RESPONSE: Thanks for checking the citations of MS. Mistakes were corrected in the new version.

REVIEWERS' COMMENTS:

Reviewer #1 (Remarks to the Author):

The authors have addressed the questions raised in the first review, providing more information in a number of areas.
Overall, the manuscript has been improved and I have also included a number of suggested edits for understanding in the attached manuscript.

From the additional information on phenotyping, it is clear that the authors are likely to be phenotyping traits which are likely to have relatively simple genetic inheritance. This would help to explain why results from the GWAS were strong and also that they are likely to correspond to major gene changes during domestication. It is also not possible to generate standard deviations or analyse data through ANOVA.

This is perhaps a weakness of the paper, but is understandable in the context of how many genotypes were used.

It would be useful for the authors to calculate heterozygosities for the lines - just how pure are the sequenced accession?
As castor produces both male and female flowers on the same plant, it seems likely that there is a reasonable level of outcrossing - this would be consistent with the rapid R2 decay observed.

Overall, the paper presents an important contribution to the genetics and trait analysis of castor bean, particularly in terms of domestication.

The greater value from the underpinning dataset will come when there are replicated field trials of subsets of these lines

Reviewer #2 (Remarks to the Author):

After revision, the quality of this manuscript is much better. I think all the questions and comments were addressed properly. Hope this manuscript will be well received by readers.

Reviewer #3 (Remarks to the Author):

The authors have addressed all the queries and the MS is now in a better form. But I would still suggest that the authors can take the help of Professional Editing Services to improve the language of the MS.

Dear editor and reviewers,

Thanks a lot for all your efforts, which helped improving the manuscript significantly. According to the further valuable suggestions, we have revised and formatted the manuscript.

Response to Peer Review Comments

Reviewer #1

COMMENTS: It would be useful for the authors to calculate heterozygosities for the lines - just how pure are the sequenced accession?
As castor produces both male and female flowers on the same plant, it seems likely that there is a reasonable level of outcrossing - this would be consistent with the rapid R2 decay observed.
RESPONSE：Heterozygosity(Hete.) could be a useful reference of population status, so we added Heterozygosity for each castor line, as a column 13 in Supplementary Data 2. Hete. Group SC and MC has a significantly higher Hete. rate than Group NC， which is consistent with R2 decay. The wild castor has a lower hete than the cultivated groups that should be frequently subject to the hybridization.

Reviewer #3

COMMENTS: I would still suggest that the authors can take the help of Professional Editing Services to improve the language of the MS.
RESPONSE：Yes, Now MS has been revised by experienced editor of Nature Publishing Group Language Editing service. The modification information was retained in changes tracking mode.
